# Deciphering the modulatory role of short-chain fatty acids in Parkinson’s disease *via* phosphorylation-dependent signaling mechanisms

**DOI:** 10.7717/peerj.20688

**Published:** 2026-01-28

**Authors:** Jiaji Liu, Ruijun Su

**Affiliations:** 1 Affiliated Hospital of Inner Mongolia Medical University, Hohhot, China; 2 Department of Laboratory Medicine, Hohhot First Hospital, Hohhot, China

**Keywords:** Parkinson’s disease, Short-chain fatty acids, Pathogenesis, Signalling pathways

## Abstract

Parkinson’s disease (PD), the world’s second most prevalent neurodegenerative disorder, is characterized by progressive neuronal degeneration mediated through intricate pathological mechanisms. Phosphorylation signaling pathways have been increasingly recognized as critical modulators in the development and progression of PD. Meanwhile, short-chain fatty acids (SCFAs), primarily produced by gut microbiota, have shown considerable neuroprotective potential by promoting autophagy, alleviating mitochondrial dysfunction, and regulating neuroinflammatory responses. Recent research suggests that SCFAs may influence the phosphorylation dynamics of key signaling pathways, including MAPKs, NF-κB, JAK/STAT, PI3K/Akt, AMPK, and Nrf2/Keap1/ARE, thereby modulating disease pathophysiology. This review aims to systematically evaluate how SCFAs modulate phosphorylation pathways to influence neuroinflammation, α-synuclein aggregation, and mitochondrial dysfunction in PD. By investigating this issue, we identify potential molecular targets and propose future research directions, offering new insighreviewts and strategies for the development of novel therapeutic and preventive interventions for PD.

## Introduction

In 2016, a research team conducted a systematic analysis of global epidemiological data, confirming that Parkinson’s disease (PD) had become the second most common neurodegenerative disorder worldwide (GBD 2016 Neurology Collaborators, 2019). By 2040, the number of individuals diagnosed with PD is projected to exceed 17 million (Dorsey et al., 2018). The hallmark symptoms of PD are motor deficits, including resting tremors, bradykinesia, and postural instability. In addition to motor symptoms, PD also presents with non-motor symptoms such as depression, constipation, and sleep disturbances (Munhoz et al., 2024). These arise due to the progressive degeneration of dopaminergic (DA) neurons in the substantia nigra pars compacta (SNpc) and the pathological aggregation of α-synuclein (Stoll et al., 2024). Evidence suggests that α-synuclein aggregation initiates well before clinical symptoms emerge. Aggregated α-synuclein in the enteric nervous system (ENS) may migrate to the brain through the dorsal motor nucleus of the vagus nerve (Espinosa-Oliva et al., 2024). This observation supports Braak’s hypothesis, which posits that PD originates in the gut (Braak et al., 2003b), It also explains why gastrointestinal disorders significantly impact patients with PD throughout their lives. For example, constipation, recognized as a prodromal symptom of PD, may precede clinical onset by up to 20 years (Bloem, Okun & Klein, 2021). Such gastrointestinal dysfunctions are frequently linked to disruptions in the gut microbiota (Kakoty et al., 2021a). Consequently, alterations in the gut microbiome are increasingly recognized as potential biomarkers for PD. To elucidate the mechanisms linking gut microbiota to PD, research has focused on their metabolic products, particularly short-chain fatty acids (SCFAs).

Recent studies have revealed that levels of SCFAs in the feces of patients with PD, as well as the SCFA-producing gut bacteria, are significantly reduced compared to healthy controls. Conversely, increased intestinal permeability in patients with PD elevates SCFA concentrations in systemic circulation, correlating with disease severity (Unger et al., 2016; Aho et al., 2021; Chen et al., 2022). At the same time, a substantial body of research indicates that SCFAs are beneficial for the health of patients with PD. SCFAs have been shown to alleviate motor symptoms in PD mouse models and enhance the expression of DA neurons (Hou et al., 2021b). SCFAs also ameliorate cognitive dysfunction in patients with PD by reducing neuroinflammation and modulating neurotransmitter balance, including gamma-aminobutyric acid (GABA) and serotonin (5-HT) (Wiley et al., 2021; Hou et al., 2021a). Importantly, SCFAs enhance intestinal motility and preserve gut barrier integrity, effectively alleviating constipation and improving the overall quality of life in patients with PD (Liao et al., 2024). These findings underscore SCFAs as key players in PD onset and progression. However, Qiao et al. (2020a) found that administering 165 mg/kg of sodium butyrate (NaB), the sodium salt of butyric acid, one of the SCFAs, for seven consecutive days exacerbated both the pathological features and clinical symptoms in an MPTP-induced PD mouse model. These conflicting findings have prompted further investigation into the specific mechanisms through which SCFAs affect PD. SCFAs reportedly bind to G protein-coupled receptors (GPCRs)/free fatty acid receptors (FFARs) and inhibit histone deacetylases (HDACs), activating intracellular phosphorylation signaling pathways (Zhang et al., 2023). Research has shown that the phosphorylation cascade regulated by SCFAs plays a critical role in modulating physiological responses, including metabolic homeostasis, immune function, and inflammatory reactions (Du et al., 2024). Especially in the central nervous system (CNS), SCFA-mediated phosphorylation signals exert neuroprotective effects by modulating pathways such as PI3K/Akt and MAPK/ERK, which are involved in neuronal survival, synaptic plasticity, and the regulation of neuroinflammation (Rai et al., 2019; Kumar et al., 2025). Phosphorylation is a key mechanism in initiating protein functions, facilitating rapid and reversible regulation of cellular processes *via* protein kinase activity (Mishra, Singh & Singh, 2024). Exploring phosphorylation signaling pathways reveals the molecular effects of SCFAs on neuronal function and survival, providing targeted therapeutic opportunities. Although phosphorylation pathways are crucial for understanding the mechanisms of SCFAs, their numerous signaling and feedback mechanisms, along with complex interactions and potential long-term effects, necessitate further research to clarify the relationships between pathways and the precise role of SCFAs in PD pathology.

This article aims to elucidate the mechanisms by which SCFAs influence PD *via* phosphorylation signaling pathways, drawing on recent research. We conduct a systematic review and analysis of SCFA metabolism, distribution, and functions in PD, emphasizing their interactions with phosphorylation signaling pathways. By investigating the regulatory roles of SCFAs in PD *via* phosphorylation processes, this study aims to offer novel insights and therapeutic strategies for future interventions.

## Survey methodology

This review is intended for researchers, clinicians, and healthcare professionals interested in understanding the role of SCFAs in PD, particularly those studying the molecular mechanisms of disease progression and potential therapeutic targets. We conducted an exhaustive and systematic literature search in the PubMed and Web of Science databases, covering publications up to 2025. The search strategy incorporated a combination of relevant keywords and Medical Subject Headings (MeSH) terms to capture all pertinent studies. The search terms included a core short-chain fatty acid (SCFA) block—(“short chain fatty acid*” OR “short-chain aliphatic acid” OR “volatile fatty acids”)—combined with each of the following concept blocks using AND: (1) “Parkinson disease” OR “Parkinson’s disease”; (2) “G protein-coupled receptor*” OR “G protein coupling receptors” OR “G-coupled protein receptor”; (3) “histone deacetylase*”; (4) “inflammatory response” OR “inflammation” OR “oxidative stress” OR “oxidant stress” OR “oxidation stress” OR “oxidative stress induced” OR “α-Synuclein” OR “gut-brain axis”; (5) “MAPKs”; (6) “NF-κB”; (7) “JAK/STAT”; (8) “PI3K/Akt”; (9) “AMPK”; and (10) “Nrf2/Keap1/ARE”. These terms were specifically designed to ensure the retrieval of all relevant literature. The inclusion criteria for the selected studies were as follows: predominantly peer-reviewed articles published between 2020 and 2025 in English, with earlier studies considered if deemed highly pertinent; all selected articles were required to be indexed in the Science Citation Index Expanded (SCIE) to ensure the highest standards of quality and scientific rigor. The exclusion criteria included case reports and conference abstracts. After retrieving the articles, the titles and abstracts were initially screened for relevance to the research topic. Studies that did not meet the criteria were excluded at this stage. The remaining articles were reviewed in full text, and only those that were directly relevant to the role of SCFAs in PD and their effects on phosphorylation signaling pathways were included. This rigorous selection process ensured that only high-quality studies were considered for inclusion in this review (Table 1).

**Table 1:** Inclusion/Exclusion criteria table.

Criteria	Inclusion	Exclusion
Study design	Studies with robust experimental designs (*e.g*., controlled clinical trials, cohort studies, systematic reviews).	Case reports, anecdotal studies, non-experimental designs (*e.g*., expert opinions, editorials).
Relevance to research topic	Studies focused on SCFAs and their role in PD, particularly on phosphorylation signaling pathways (MAPKs, NF-κB, JAK/STAT, PI3K/Akt, AMPK, Nrf2/Keap1/ARE).	Studies not related to SCFAs, PD, or phosphorylation signaling.
Scientific rigor	Only peer-reviewed articles indexed in the SCIE, ensuring high-quality, scientifically rigorous work.	Non-peer-reviewed studies, articles not indexed in SCIE, or articles published in low-impact journals.
Sample size and statistical methods	Studies with adequate sample sizes and clear reporting of statistical methods, ensuring the robustness of findings.	Studies with insufficient sample sizes, lack of statistical analysis, or unclear reporting of methods.
Language	Articles published in English.	Articles not in English or with insufficient translation accuracy.
Publication date	Articles published between 2020 and 2025, with earlier studies included if deemed highly relevant.	Studies published before 2020, unless highly pertinent to the topic of SCFAs and PD.

**Note:**

MAPKs, Mitogen-activated protein kinases; NF-κB, nuclear factor kappa B; JAK/STAT, Janus kinase/signal transducer and activator of transcription; PI3K/Akt, phosphatidylinositol 3-kinase/protein kinase B; AMPK, AMP-activated protein kinase; Nrf2, nuclear factor erythroid 2-related factor 2; Keap1, Kelch-like ECH-associated protein 1; ARE, antioxidant response element; SCFAs, short-chain fatty acids; PD, Parkinson’s disease; SCIE, science citation index expanded.

## Mechanism of scfas

### Metabolic pathway of SCFAs

SCFAs, saturated fatty acids with fewer than six carbon atoms, are primarily derived from the anaerobic fermentation of indigestible carbohydrates by gut microbiota, including Bacteroides and Prevotella, with these fatty acids being predominantly released in the gut and reaching their highest concentrations in the colon (Li et al., 2022; Hays, Pfaffinger & Ryznar, 2024). Studies employing gas chromatography (GC) and high-performance liquid chromatography (HPLC), which are considered the gold standards for quantifying SCFAs, have demonstrated that SCFAs are most concentrated in the proximal colon, with concentrations ranging from 70 to 140 mmol/L. The primary SCFAs include acetate (C2), propionate (C3), and butyrate (C4), which occur in an approximate ratio of 60:25:15 (Cummings et al., 1987; Meesters et al., 2007; Hays, Pfaffinger & Ryznar, 2024). Approximately 90% of SCFAs are rapidly absorbed by intestinal cells due to their high volatility and water solubility (Roediger, 1980). A small fraction of SCFAs in neutral form crosses the intestinal epithelial barrier *via* passive diffusion, whereas the majority, in their ionized state, are actively transported into colonic cells through epithelial membrane receptors, including H^+^-dependent monocarboxylate transporter-1 (MCT1, SLC16A1), Na^+^-coupled monocarboxylate transporter-1 (SMCT1, SLC5A8), and the basolateral transporters MCT4 (SLC16A3) and MCT5 (SLC16A4) (Gill et al., 2005; Kaji et al., 2015; Stumpff, 2018). Inside the cells, SCFAs, particularly butyrate, undergo β-oxidation and the tricarboxylic acid cycle to supply 60–70% of the energy required by colonic cells and contribute approximately 10% of the energy needed for daily human physiological demands (Guo et al., 2023b). Following absorption, SCFAs enter systemic circulation *via* the hepatic portal vein. However, due to the first-pass metabolism in the liver, the concentration of acetate in systemic circulation is only 100–200 μM, while propionate and butyrate levels are even lower, at 1–15 μM (Moreau et al., 2003; Meesters et al., 2007). SCFAs bypassing the liver can cross the blood-brain barrier (BBB) to reach the brain, where the average concentrations of propionate and butyrate are 18.8 and 17.0 pmol/mg, respectively (Silva, Bernardi & Frozza, 2020). Notably, the concentrations of SCFAs used in *in vitro* experiments often exceed the physiological levels found in the human brain. This discrepancy represents an important limitation when interpreting experimental findings, as supraphysiological exposure to SCFAs may elicit effects and activate signaling pathways that are unlikely to be engaged at brain-relevant concentrations *in vivo*.

### SCFAs signaling pathways

**Figure 1:**
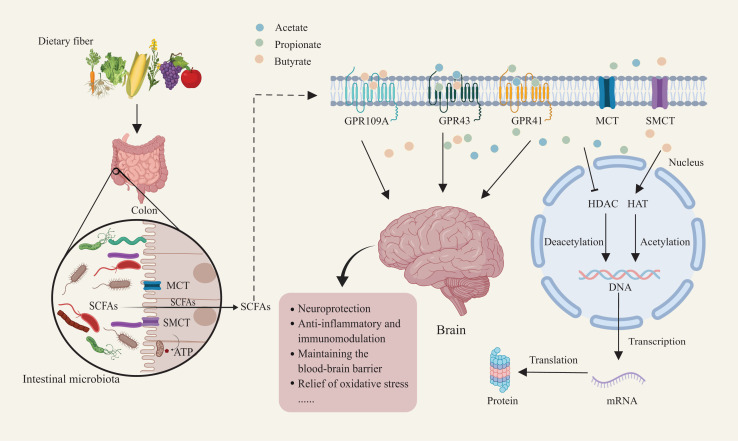
Mechanisms of dietary fiber metabolism and SCFAs in neuroprotection. Gut microbiota metabolize dietary fiber to produce SCFAs such as acetate, propionate, and butyrate. SCFAs enter host cells through short-chain fatty acid transporters. Inside the cell, SCFAs activate receptors like GPR109A and GPR43/GPR41. SCFA receptor activation facilitates deacetylation and acetylation, regulating nuclear transcription factor activity. This, in turn, influences DNA and mRNA expression, ultimately modulating protein synthesis and translation. This mechanism supports neuroprotection, anti-inflammatory and immunomodulatory functions, while maintaining blood-brain barrier integrity and reducing oxidative stress. Created with MedPeer (medpeer.cn).

Extensive research has identified two primary mechanisms by which SCFAs influence biological functions in the human body. First, SCFAs interact with GPCRs, primarily GPR41, GPR43, and GPR109A, which play critical roles in regulating host immune and metabolic responses (Fig. 1) (Du et al., 2024). Second, SCFAs directly inhibit HDAC activity, which enables precise chromatin remodeling and regulation of gene expression. This mechanism profoundly influences gene expression across various cell types, enhancing their adaptability to environmental changes (Du et al., 2024). In the context of Parkinson’s disease, these two signaling modes provide a mechanistic framework for interpreting experimental and clinical data on gut microbiota–derived SCFAs. Using an α-synuclein–overexpressing mouse model, Sampson et al. (2016) demonstrated that gut microbiota and their SCFA metabolites are necessary to elicit PD-like motor deficits and neuroinflammation: germ-free or antibiotic-treated mice show reduced α-synuclein aggregation and microglial activation, whereas recolonization with complex microbiota or oral SCFA supplementation reinstates these pathological and behavioral phenotypes. Together with human studies reporting altered SCFA profiles, impaired gut barrier integrity and dysregulated free fatty acid receptor expression in PD (Unger et al., 2016; Aho et al., 2021; Chen et al., 2022; Liao et al., 2024), these findings support the view that GPCR- and HDAC-mediated SCFA signaling constitutes a key molecular interface along the gut–brain axis, which will be further elaborated in the following sections.

#### Interaction with GPCRs

GPR43, also referred to as FFAR2, conjugates with Gi/o to mediate its function outside the gut and interacts with Gq in the gut (Koh et al., 2016). The main ligands for GPR43 are acetate, propionate, and, to a lesser extent, butyrate, with acetate being the most specific activator and propionate exhibiting the highest activation efficiency (Tan et al., 2014). With an effective concentration (EC50) of 250–500 μM for acetate and propionate, GPR43 remains nearly continuously active in the intestinal lumen, mediating biological functions (Koh et al., 2016). GPR43 is widely expressed in immune cells, including lymphocytes, neutrophils, and monocytes, indicating its key role in mediating SCFAs’ effects on immune responses (Layden et al., 2013). Research shows that SCFAs, *via* GPR43, regulate oligomerization domain-like receptors 3 (NLRP3) inflammasome activity, promote Treg differentiation, and suppress pro-inflammatory cytokines such as TNF-α and IL-6, highlighting their therapeutic potential in managing inflammatory diseases (Hou et al., 2021a; Wang et al., 2023; Fang et al., 2024). Recent research shows that while GPR43 suppresses inflammatory gene expression in microglia under healthy conditions, it fails to effectively regulate microglial activation during inflammation, and the absence of GPR43 expression in microglia suggests that SCFAs may exert indirect effects on microglia through multiple pathways, including the peripheral immune system, highlighting the need for further investigation of GPR43’s role in various disease contexts and physiological states (Caetano-Silva et al., 2023, 2024).

In contrast, GPR41/FFAR3, although GPR43 and GPR41 belong to the same receptor family, they exhibit significant differences in protein structure, leading to distinct affinities for various SCFAs (Brown et al., 2003). For instance, GPR41 exclusively couples with Gi proteins, demonstrating the highest sensitivity to propionate, followed by butyrate, with significantly lower sensitivity to acetate (Koh et al., 2016). GPR41 is predominantly expressed in sympathetic ganglia, where it plays a key role in regulating sympathetic nervous activity (Kimura et al., 2011). By interacting with SCFAs, GPR41 facilitates the transmission of neural signals from the periphery to the central nervous system, with propionate activating GPR41 in neurons near the portal vein and transmitting signals through vagal and spinal pathways to elicit physiological responses, such as protecting the nervous system, regulating the immune system, and maintaining gut homeostasis (Zhang et al., 2023). Additionally, osteocalcin modulates gut microbiota to elevate propionate levels, which then activates GPR41 in the ENS to prevent motor dysfunction and dopamine neuron loss in PD mouse models (Hou et al., 2021b). Other studies indicate that butyrate-mediated activation of GPR41 mitigates salsolinol-induced dopaminergic cytotoxicity in SH-SY5Y cells (Getachew et al., 2020). Extensive research suggests that under SCFA stimulation, both GPR41 and GPR43 promote the secretion of glucagon-like peptide-1 (GLP-1) and peptide YY (PYY) from intestinal L-cells, exerting neuroprotective effects in PD models, although some studies propose that SCFA-induced GLP-1 secretion occurs independently of GPR41 and GPR43, with the underlying mechanism remaining unclear (Psichas et al., 2015; Liu et al., 2017; Christiansen et al., 2018).

GPR109A (also referred to as HCAR2) exclusively couples with Gi proteins and is predominantly expressed in adipocytes and immune cells (Chai, Digby & Choudhury, 2013). Although niacin (vitamin B3) is a potent agonist, physiological concentrations of niacin are typically insufficient to significantly activate GPR109A under normal conditions (Wanders, Graff & Judd, 2012). In contrast, the EC50 for butyrate activation of GPR109A is approximately 1 mM, which, despite its low affinity, supports stable and sustained interactions (Kasubuchi et al., 2015). Activated GPR109A plays a crucial role in regulating lipolysis and immune inflammation, but its expression declines with age, potentially increasing the risk of fat accumulation and metabolic disorders in older adults (Wang et al., 2020; Lee et al., 2020). Moreover, NaB-induced activation of GPR109A strengthens gut barrier integrity, suppresses systemic inflammation, and mitigates PD pathology (Xu et al., 2022b). The overlapping distribution and functional similarities of GPR43, GPR41, and GPR109A create some uncertainty regarding their specific roles. However, each receptor contributes uniquely to physiological processes. GPR43 is primarily involved in regulating immune responses and inflammation, GPR41 plays a key role in gut-brain signaling and autonomic functions, and GPR109A is critical for lipid metabolism and maintaining gut barrier integrity (Huang et al., 2020; Inoue, Tsujimoto & Kimura, 2014; Xu et al., 2022b). These distinct functions underscore the need for further research to clarify their interactions and mechanisms in various disease contexts.

#### Inhibition of HDACs

Histone acetylation is a key regulator of gene expression, dynamically controlled by HATs and HDACs (Slaughter et al., 2021). HDACs are classified based on their cofactor dependency into NAD^+^-dependent (SIRT1-7) and Zn^2+^-dependent (HDAC1-11) categories, and further divided into four classes (I–IV) (Leite et al., 2022). Aberrant HDAC activation is implicated in the pathogenesis of neurodegenerative diseases, particularly PD, where it may lead to the overexpression of the alpha-synuclein (SNCA) gene, causing abnormal α-synuclein aggregation (van Heesbeen & Smidt, 2019; Toker et al., 2021). Consequently, HDAC inhibitors (HDACis) have emerged as a promising therapeutic approach to regulate histone acetylation.

Numerous studies indicate that SCFAs function as HDAC inhibitors, facilitating histone hyperacetylation and exerting neuroprotective effects (Stilling et al., 2016). Of the SCFAs, butyrate is the most effective HDAC inhibitor, selectively inhibiting Class I HDACs with up to 80% efficiency, whereas propionate achieves around 60% inhibition (Guo et al., 2023b). Both SCFAs can inhibit class I/II HDACs and may indirectly modulate class III (Sirtuin) activity by altering intracellular NAD^+^ levels (Wu et al., 2012). Chen et al. (2007) found that treatment with 1.2 mM NaB for 12 h significantly increased histone H3 acetylation and induced significant apoptosis in microglial cells, which in turn suppressed pro-inflammatory cytokine secretion (*e.g*., TNF-α), thereby mitigating neuroinflammation. Furthermore, the team observed that transcription of neurotrophic factors such as glial cell line-derived neurotrophic factor and brain-derived neurotrophic factor is positively correlated with histone acetylation levels, underscoring the potential of HDACis to exert neuroprotection by modulating astrocyte function (Wu et al., 2008). SCFAs improve rotenone-induced motor dysfunction in Drosophila through mechanisms involving tyrosine hydroxylase (TH) expression, dopamine levels, and oxidative stress pathways, and in Sin3A-deficient Drosophila (Sin3Alof), where Sin3A regulates HDAC activity, rotenone-induced mortality and motor deficits were partially reversed (St. Laurent, O’Brien & Ahmad, 2013). Studies further indicate that co-administration of SCFAs with the autophagy inducer trehalose in a PFFα-syn-induced PD model significantly reduces pro-inflammatory cytokine levels while enhancing dopamine production and histone H3 acetylation, providing novel insights into the therapeutic applications of SCFAs in PD and highlighting their neuroprotective effects through epigenetic regulation and autophagy modulation (Kakoty et al., 2021b). Nevertheless, combined *in vivo* and *in vitro* studies suggest that SCFAs may induce histone hyperacetylation at the promoter of oxidative stress-sensitive PKCδ, increasing its transcriptional activity and leading to excessive PKCδ activation, which disrupts intracellular signaling balance and exacerbates neuronal damage and death (Jin et al., 2014). Thus, the multifaceted roles of SCFAs require careful evaluation to minimize potential neurotoxicity in therapeutic contexts.

## Pathogenesis of parkinson’s disease

### Abnormal aggregation of α-synuclein

α-synuclein, a 140-amino-acid soluble monomeric protein, predominantly localizes to synaptic terminals, where it facilitates vesicle transport and neurotransmitter release (Schweighauser et al., 2020). During the pathological progression of PD, mutations in the SNCA gene and stress conditions drive α-synuclein to shift from a soluble α-helix to an insoluble β-sheet conformation, forming oligomers (Mehra, Sahay & Maji, 2019). Growing evidence suggests that early-stage oligomers are the primary neurotoxic agents, impairing synaptic function, damaging organelles such as mitochondria and lysosomes, and ultimately causing neuronal dysfunction and death, with early synaptic dysfunction being a critical step in PD pathogenesis, while these oligomers also exert direct toxicity and promote the aggregation of normal α-synuclein, leading to insoluble fibrillar deposits that eventually form classic Lewy bodies (Braak et al., 2003a; Alam et al., 2019). Abnormal α-synuclein aggregation induces neuronal dysfunction, mitochondrial impairment, and inflammatory responses, while promoting its pathological spread through inter-neuronal transmission (Lehtonen et al., 2019). Early studies found that PD patients receiving fetal neuron transplants to restore DA neurons developed Lewy bodies in the transplanted neurons, raising significant concerns (Kordower et al., 2008; Li et al., 2008). Subsequent studies using α-synuclein-overexpressing animal models demonstrated its prion-like behavior, spreading pathologically between neurons and causing further cellular damage (Luk et al., 2012; Recasens et al., 2018; Tarutani & Hasegawa, 2019). This mechanism of “intercellular propagation” supports Braak et al.’s (2003a) staging theory, highlighting the spatial and temporal progression of PD pathology, and offers a molecular explanation for the early emergence of non-motor symptoms in PD. The formation of α-synuclein aggregates represents a pivotal event in PD pathology. However, the role of Ser129 phosphorylation (Ser129P) in this process remains unclear. Ser129 phosphorylation is extensively observed in α-synuclein within Lewy bodies and Lewy neurites in the brains of patients with PD, suggesting a strong association with aggregate formation (Anderson et al., 2006). If Ser129P directly drives pathological α-synuclein aggregation, its elevated levels should enhance neurodegeneration and cell death; however, animal studies do not support this hypothesis, as although S129D mutants exhibited slightly accelerated degeneration in models such as Drosophila and yeast, the overall data did not indicate significant neurodegenerative changes (Chen & Feany, 2005; Gitler et al., 2007; Soper et al., 2008). Traditional perspectives often overlook the physiological roles of Ser129P, viewing it solely as a pathological marker, while recent research suggests that Ser129P acts as a dynamic regulator with potential protective effects in early disease stages, modulating α-synuclein’s synaptic localization and interactions with other synaptic proteins to inhibit synaptic transmission, regulate neurotransmitter release, and maintain neuronal activity and synaptic function (Parra-Rivas et al., 2023). Furthermore, Ser129 phosphorylation may influence the aggregation process by stabilizing α-synuclein oligomers, potentially promoting the transition from oligomeric to fibrillar forms under pathological conditions (Parra-Rivas et al., 2023). Therefore, Ser129P should not be viewed merely as a direct pathological inducer. Investigating how Ser129P regulates α-synuclein through neuronal activity and protein-protein interactions may provide insights into the pathogenesis of PD and other synucleinopathies, paving the way for future therapeutic strategies.

### Mitochondrial dysfunction and oxidative stress

Mitochondrial dysfunction represents a key pathological mechanism in the early stages of PD. It is closely associated with synaptic damage, overproduction of reactive oxygen species (ROS), disruption of calcium homeostasis, imbalance of neuronal microtubule acetylation and decreased intracellular adenosine triphosphate (ATP) synthesis (Nicoletti et al., 2021; Naren et al., 2023b). In PD models induced by chemical agents like MPTP and rotenone, these compounds impair mitochondrial complex I activity to exert their toxic effects. These models demonstrate a direct link between mitochondrial dysfunction and PD neuropathology, offering vital experimental insights into the disease mechanisms (Mani et al., 2021). Extensive research reveals that mitochondrial dysfunction in PD extends beyond impaired electron transport chain (ETC) activity. It also encompasses severe disruptions in mitochondrial structure and dynamics. Damage to mitochondrial architecture decreases ATP production, impairs neuronal energy metabolism, and accelerates neurodegeneration (Nicoletti et al., 2021; Naren et al., 2023a; Tryphena et al., 2023). During PD progression, the abnormal aggregation of α-synuclein induces the translocation of dynamin-related protein 1 (Drp1), a key regulator of mitochondrial fission, disrupting mitochondrial structure and impairing the dynamic fusion-fission balance essential for maintaining mitochondrial function (Krzystek et al., 2021). Mitophagy, the selective removal of damaged mitochondria, is essential for maintaining mitochondrial health and minimizing energy inefficiency, with PINK1 and the E3 ubiquitin ligase Parkin serving as key regulators; under excessive stress, mitochondria in PD lose membrane potential, leading to PINK1 accumulation on their outer membranes and subsequent ubiquitin phosphorylation, which recruits and activates Parkin through PINK1 phosphorylation, ultimately resulting in the ubiquitination and degradation of mitochondrial surface proteins (Narendra & Youle, 2024). However, α-synuclein aggregates severely impair Parkin function, and under α-synuclein influence, Parkin levels decline significantly, exacerbating mitochondrial toxicity (Sharma et al., 2024). Extensive genetic studies emphasize the critical role of mitochondria in PD pathology, with genes such as PRKN, PINK1, and LRRK2 implicated in mitochondrial bioenergetics disruption and quality control impairment; for instance, PINK1 and PRKN are essential for mitophagy regulation, while mutations in LRRK2, particularly the Gly2019Ser mutation, are strongly associated with PD, interfering with mitotic processes and mitochondrial dynamics, ultimately leading to extensive structural and functional mitochondrial collapse (Singh & Ganley, 2021). These findings clearly demonstrate the direct effects of genetic mutations on mitochondrial function, emphasizing the intricate and crucial role of genetic factors in PD pathology.

In various animal models of PD, the accumulation of ROS, including superoxide, hydrogen peroxide, and hydroxyl radicals, is frequently observed, providing strong evidence for the pivotal role of oxidative stress in neuronal degeneration (Ding et al., 2018). At moderate levels, ROS regulate cellular signaling and support normal physiological functions, but excessive ROS accumulation damages essential cellular components such as lipids, proteins, and DNA, leading to oxidative damage and triggering a cascade of pathological reactions (Umeno, Biju & Yoshida, 2017). DA neurons in the substantia nigra of PD patients are highly susceptible to oxidative stress due to their unique morphology and high energy requirements, with oxidative stress directly endangering these neurons and disrupting their complex network connections, and this susceptibility stemming from an imbalance between pro-oxidant and antioxidant systems, a key factor in PD progression (Ren et al., 2017). Consequently, oxidative stress is considered a major contributor to neuronal degeneration in PD pathology. This phenomenon is closely associated with mitochondrial dysfunction. Mitochondrial damage and ETC disruption—especially complex I (NADH dehydrogenase) inhibition—are primary sources of intracellular ROS overproduction in PD (Malpartida et al., 2021). Reduced electron transport efficiency causes ROS accumulation, which directly damages mitochondrial DNA (mtDNA) within mitochondria. mtDNA damage further impairs mitochondrial function, especially ETC activity, perpetuating a vicious cycle of “oxidative stress and mitochondrial dysfunction” (Picca et al., 2020).

### Inflammation and immune response

**Figure 2:**
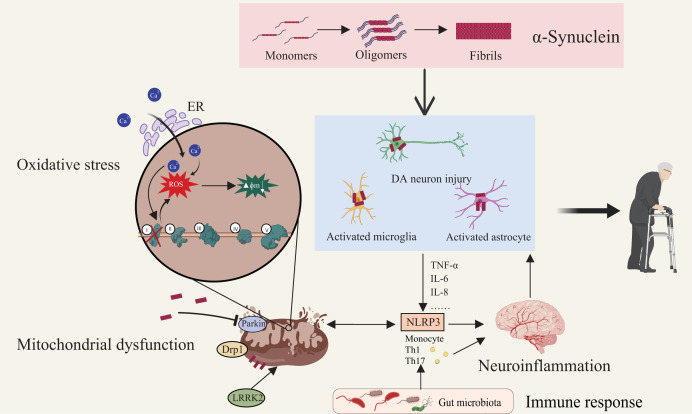
Multifactorial interactions driving Parkinson’s disease pathogenesis. Aggregation of α-synuclein initiates its transition from monomeric to oligomeric and fibrillar forms, leading to activation of astrocytes and microglia and subsequent DA neuronal injury. Mitochondrial dysfunction—characterized by membrane depolarization, increased permeability, structural fragmentation, and respiratory chain defects—compromises cellular energy metabolism and intensifies oxidative stress. Defective mitophagy exacerbates mitochondrial damage, elevating cytosolic Ca^2+^ and generating ROS which drive apoptotic cascades. Proteins such as Drp1 and LRRK2 regulate mitochondrial morphology and neuronal viability. Notably, neuroinflammation is both triggered and sustained by these pathological events. Immune responses, modulated by microbiota-derived metabolites and peripheral immune activation, converge on microglia and astrocytes, amplifying central inflammation *via* pathways such as NLRP3 inflammasome signaling. This highlights the gut–brain–immune axis as a critical contributor to PD pathogenesis. Created with MedPeer (medpeer.cn).

Since the 1980s, the discovery of microglial activation and elevated pro-inflammatory cytokines in post-mortem PD brains has established neuroinflammation as a crucial component of PD pathology. The role of microglia in PD has been well-documented and extensively validated (Morris et al., 2024). As the primary innate immune cells of the CNS, activated microglia release significant amounts of pro-inflammatory cytokines and chemokines, including TNF-α, IL-6, and IL-8, with persistent microglial activation during disease progression serving as a source of inflammatory responses and directly contributing to the damage of DA neurons (Zhang, Tang & Illes, 2024). The hallmark pathological protein in PD, α-synuclein, is closely associated with neuroinflammation, and its interaction with CNTFR-α on DA neurons activates the JAK-STAT3 signaling pathway, further amplifying inflammation and microglial activation, thereby creating a self-perpetuating vicious cycle (Liu et al., 2010). Additionally, the abnormal aggregation of α-synuclein not only leads to neuronal damage but also serves as a key trigger for immune system activation, as α-synuclein directly interacts with toll-like receptors on microglia and astrocytes, initiating the downstream activation of the NLRP3 inflammasome and various inflammatory mediators, ultimately resulting in caspase-1-dependent mitochondrial damage and subsequent DA neuronal injury (Soraci et al., 2023; Brash-Arias et al., 2024). Notably, the NLRP3 inflammasome serves as a critical bridge between mitochondrial dysfunction and neuroinflammation, playing a pivotal role in this pathological interplay (Khot et al., 2022). Mitochondrial dysfunction in PD patients is associated with abnormal calcium (Ca^2+^) accumulation, impaired mitogen-activated protein kinase (MAPK) activity, and mitochondrial antiviral signaling protein (MAVS) alterations, which collectively overactivate the NLRP3 inflammasome, promoting the release of pro-inflammatory cytokines such as IL-1β and IL-18 and thereby exacerbating neuroinflammation (Gurung, Lukens & Kanneganti, 2015). Therefore, the NLRP3 inflammasome is both a consequence of mitochondrial dysfunction and an amplifier of neuroinflammation, establishing a vicious cycle between the two that accelerates the pathological progression of PD. Recent advances further reveal that CNS inflammatory circuits are intimately linked to gut-mediated immune modulation, as microbiota-derived metabolites and barrier perturbations reshape peripheral immune states and transmit pro-inflammatory signals to the brain, thereby embedding gut–immune communication within the broader neurodegenerative cascade of PD (Sampson et al., 2016; Mou et al., 2022). Increasing evidence underscores the critical role of the peripheral immune system in PD pathology, as peripheral blood in PD patients shows elevated levels of pro-inflammatory monocytes and T-cell subsets, with monocytes exhibiting a classical pro-inflammatory phenotype and T-cells primarily differentiating into Th1 and Th17 subtypes, and these immune alterations being strongly linked to increased pro-inflammatory cytokine expression (Contaldi, Magistrelli & Comi, 2022). Genetic variations in the HLA region have been shown to significantly correlate with PD risk. Studies indicate a strong link between HLA risk alleles and α-syn-specific T-cell responses, highlighting the pivotal role of the adaptive immune system in PD pathogenesis (Sulzer et al., 2017). Although current research offers valuable insights into immune-inflammatory mechanisms in PD, understanding longitudinal immune changes and their effects across disease stages remains a significant challenge for developing precise therapeutic strategies (Fig. 2).

## Scfas mediate multiple phosphorylation signaling pathways affecting pd

Phosphorylation is a fundamental mechanism that enables cells to detect and respond to external signals. It plays a pivotal role in signal transduction by adding phosphate groups to specific amino acid residues on proteins, thereby regulating their activity, localization, and interactions with other molecules, ultimately modulating the activation or inhibition of signaling pathways (Ardito et al., 2017). Recent studies highlight the critical role of phosphorylation signaling pathways in PD pathogenesis, particularly in essential biological processes like neuronal apoptosis, autophagy, stress responses, mitochondrial function, and protein degradation (Jin et al., 2024). SCFAs regulate key proteins in these pathways, enhancing neuronal survival, reducing inflammatory responses, and alleviating oxidative stress, a process essential for protecting DA neurons in the substantia nigra in PD by modulating phosphorylation signaling pathways (Table 2), thereby potentially reducing neuronal damage and slowing disease progression (Duan et al., 2023).

**Table 2:** SCFAs influence PD through multiple phosphorylation signaling pathways.

Signal pathway	Mechanism of SCFA action	Potential applications	References
MAPKs	Activates ERK1/2 and p38 MAPK *via* GPR41 and GPR43; Regulates chemokine and cytokine production.	Modulates leukocyte recruitment, impacts PD pathology, and alleviates neuroinflammation.	Bayazid et al. (2021), Kim et al. (2013), Yshii et al. (2022)
NF-κB	Regulates GPR109A receptor and inhibits HDAC3 activity to prevent overactivation.	Reduces inflammatory responses, offering neuroprotection.	Hu et al. (2024), Xu et al. (2022b)
JAK/STAT	Suppresses excessive activation of JAK2/STAT3; Enhances iNOS and NO production in specific cells.	Mitigates oxidative stress, reduces pro-inflammatory cytokines, and supports potential PD therapies.	Ji et al. (2023), Stempelj et al. (2007), Ying, Wei & An (2021)
PI3K/Akt	Reduces neuronal apoptosis; promotes autophagy; Lowers oxidative stress.	Protects DA neurons and slows disease progression.	Qiao et al. (2020b), Wu et al. (2022), Zhou et al. (2021)
AMPK	Activates AMPK to improve mitochondrial function; Enhances mitophagy; alleviates oxidative stress.	Offers pathways to ameliorate cognitive dysfunction and maintain mitochondrial health in PD.	Watchon et al. (2024), Li et al. (2022)
Nrf2/Keap1/ARE	Induces Nrf2 nuclear translocation *via* HDAC inhibition; Enhances antioxidant capacity.	Regulates oxidative stress and neuroinflammation while protecting BBB integrity.	Fock & Parnova (2023), Guo et al. (2020), Wu et al. (2018)

**Note:**

MAPKs, Mitogen-activated protein kinases; ERK1/2, extracellular signal-regulated kinase 1/2; GPR41, G protein-coupled receptor 41; GPR43, G protein-coupled receptor 43; PD, Parkinson’s disease; NF-κB, nuclear factor kappa B; GPR109A, G protein-coupled receptor 109A; HDAC3, Histone deacetylase 3; JAK/STAT, Janus kinase/signal transducer and activator of transcription; iNOS, inducible nitric oxide synthase; NO, nitric oxide; PI3K/Akt, phosphatidylinositol 3-kinase/protein kinase B; DA, dopaminergic; AMPK, AMP-activated protein kinase; Nrf2, nuclear factor erythroid 2-related factor 2; Keap1, Kelch-like ECH-associated protein 1; ARE, antioxidant response element; BBB, blood-brain barrier.

### MAPKs signaling pathway

MAPKs, crucial intracellular signaling molecules from the serine/threonine protein kinase family, play essential roles in various cellular processes by responding to external stimuli such as growth factors, stress, inflammation, and cellular damage, with these signals being transmitted to the nucleus to regulate cell proliferation, differentiation, apoptosis, and survival, underscoring the vital role of MAPKs in adapting to environmental changes and regulating cellular fate (Guo et al., 2023a). The MAPK signaling pathway functions through a cascade of sequentially activated kinases, encompassing subtypes like extracellular signal-regulated kinase (ERK), c-Jun N-terminal kinase (JNK), and p38 MAPK. In PD, aberrant activation of these pathways is strongly linked to the loss of DA neurons as well as oxidative stress and inflammatory responses in neurons (Gil-Martinez et al., 2020). The ERK pathway, a key branch of the MAPK signaling cascade, primarily regulates cell proliferation and survival, with research indicating that the ERK1/2 signaling pathway protects DA neurons from neurotoxins like MPP+ and mitigates neuronal damage in PD by modulating gene expression associated with cell survival and apoptosis (Li et al., 2023b). However, in a PD mouse model induced by 6-hydroxydopamine (6-OHDA), excessive activation of the ERK pathway was observed, with this hyperactivation being modulated by the cAMP/PKA pathway in direct pathway neurons (dSPNs) and calcium signaling mediated by AMPA receptors in indirect pathway neurons (iSPNs), ultimately disrupting motor control through distinct molecular mechanisms and contributing to the characteristic motor symptoms of PD (Mariani et al., 2019). Thus, the ERK pathway exhibits dual roles in PD: it provides neuroprotection under normal conditions but exacerbates disease progression when abnormally activated. This complexity highlights the need for precise timing and method selection in therapeutic interventions. The JNK pathway serves as a critical regulator of intracellular stress responses in the MAPK family. It involves small G proteins, including Rac and Cdc42, that activate downstream kinases, resulting in JNK phosphorylation and activation (Coso et al., 1995). Activated JNK translocates to the nucleus, where it activates transcription factors that induce the expression of pro-apoptotic genes, resulting in progressive neuronal loss (Li et al., 2024). Furthermore, JNK-mediated phosphorylation activates the transcription factor forkhead box protein O1 (FoxO1), a critical mediator of oxidative stress and apoptosis in various cell types, including neurons (Liu et al., 2020). Additionally, the JNK pathway promotes neuroinflammation by activating multiple pro-inflammatory factors, and studies have shown that inhibiting this pathway reduces oxidative stress, inflammation, and neuronal damage associated with rotenone-induced neurodegeneration (Vasudevan Sajini et al., 2024). Similar to JNK, the p38 MAPK pathway is primarily activated by oxidative stress and inflammation-related stimuli, regulating inflammatory responses at both transcriptional and translational levels as a key mediator of neuroinflammation, while also modulating the expression of inflammatory cytokines and stress-response genes through multiple downstream targets (Hwang et al., 2016). Additionally, studies suggest that excessive activation of the p38 MAPK pathway in PD not only exacerbates inflammation but also leads to mitochondrial dysfunction and apoptosis pathways, making cells more susceptible to oxidative stress and further promoting neuronal apoptosis and inflammation (Chen et al., 2018). In summary, activation of the MAPK pathways plays a critical role in driving oxidative stress and neuroinflammation, accelerating the neurodegenerative progression of PD.

The regulation of MAPKs pathways by SCFAs primarily depend on GPCRs. When SCFAs interact with intestinal epithelial cells *via* GPR41 and GPR43, they activate the ERK1/2 and p38 MAPK signaling pathways, leading to the production of chemokines and cytokines that regulate leukocyte recruitment and effector T-cell activation, with activated T cells disrupting the BBB or directly infiltrating the brain, thereby initiating neuroinflammation and contributing to PD pathology (Kim et al., 2013; Yshii et al., 2022). However, the concentrations of SCFAs in the brain are much lower than those typically used in *in vitro* studies, which may limit the direct applicability of these findings to physiological conditions. Nevertheless, SCFAs exhibit complex and dual effects, depending on their concentration and context. In a neuroinflammation model using TNF-α-stimulated SH-SY5Y neuroblastoma cells, sodium butyrate (NaB) reduced p38 phosphorylation and decreased the expression of inflammatory markers, including inducible nitric oxide synthase (iNOS) and cyclooxygenase-2 (COX-2), thereby attenuating oxidative stress and inflammation and contributing to neuronal protection (Bayazid et al., 2021). Therefore, we hypothesize that the pro-inflammatory effects of SCFAs may contribute negatively during the early or progressive stages of PD. However, following neuronal damage and neuroinflammation, SCFAs may exert neuroprotective effects by inhibiting MAPK pathways. The underlying mechanisms remain to be elucidated, but targeting SCFA signaling pathways could offer a more precise therapeutic strategy.

### NF-κB signaling pathway

The Nuclear Factor kappa B (NF-κB) signaling pathway is a versatile regulatory system widely present in various nervous system cell types, including neurons, oligodendrocytes, microglia, and astrocytes, primarily regulating immune and inflammatory responses while also controlling cell survival and apoptosis (Cildir, Low & Tergaonkar, 2016). NF-κB comprises protein dimers, including p50, p52, p65/RelA, c-Rel, and RelB, with subunit combinations providing specific regulatory functions in different cell types (Salles, Romano & Freudenthal, 2014). Under normal physiological conditions, NF-κB is inhibited by the IκB protein complex, which retains it in the cytoplasm; however, in the pathological environment of PD, external stimuli activate cell surface receptors, triggering a signaling cascade through adaptor proteins that leads to the activation of the IκB kinase (IKK) complex, which phosphorylates IκB, causing its degradation and the release of NF-κB dimers that translocate to the nucleus to initiate the transcription of pro-inflammatory genes (Yshii et al., 2022). Previous studies have shown that persistent neuroinflammation and α-synuclein aggregates in PD activate microglia and elevate pro-inflammatory cytokine levels, leading to excessive activation of the NF-κB pathway, which in turn triggers further pro-inflammatory cytokine release, creating a vicious cycle that exacerbates neuronal damage and death (Dolatshahi et al., 2021; Leandrou et al., 2024). NF-κB activation also plays a critical role in the apoptosis of DA neurons in PD. Under prolonged neuronal stress and oxidative conditions, NF-κB interacts with apoptosis-related proteins like p53 and Bcl-2-associated X protein (Bax), enhancing apoptotic signaling and causing irreversible neuronal damage (Chauhan et al., 2016). While the detrimental effects of NF-κB in PD are well-documented, its complexity and dual roles warrant consideration. Some evidence suggests that NF-κB may have neuroprotective effects under specific conditions, as TNF-α, through the NF-κB pathway, activates downstream complexes such as TNFR1-Associated Death Domain Protein and TNF Receptor-Associated Factor 2 (TRAF2) *via* TNF receptors, facilitating NF-κB translocation to the nucleus and inducing the expression of anti-apoptotic genes, thereby counteracting apoptosis triggered by cellular stress (Heyninck & Beyaert, 2001). Receptor-interacting proteins (RIPs) and TRAF2 regulate IKK activation, suppress apoptotic signaling, and promote neuronal survival (Zhang et al., 2009). These mechanisms are essential for neuronal development, synaptic plasticity, and acute stress responses, maintaining normal central nervous system function. While the pro-inflammatory and pro-apoptotic roles of the NF-κB pathway in PD are well-researched, its dual functions also suggest potential neuroprotective mechanisms. However, drugs directly targeting NF-κB have demonstrated limited efficacy in PD treatment due to off-target toxicity and poor specificity (Gray et al., 2014). Therefore, SCFAs exert a precise regulatory effect by not only modulating GPR109A receptors to inhibit NF-κB activity but also suppressing HDAC3-mediated acetylation of STAT1 and NF-κB p65, thereby preventing the nuclear translocation of NF-κB p65 and interrupting the amplification of inflammatory cascades (Xu et al., 2022b; Hu et al., 2024). Through these mechanisms, SCFAs not only significantly attenuate inflammatory responses but also offer a targeted immunoregulatory strategy, highlighting their promising therapeutic potential for neuroprotection and anti-inflammatory interventions.

### JAK/STAT signaling pathway

The Janus kinase/signal transducer and activator of transcription (JAK/STAT) signaling pathway is a key mediator of cytokine and growth factor responses, playing a critical role in immune regulation, cell growth, differentiation, and apoptosis, with the Janus kinase (JAK) family (JAK1, JAK2, JAK3, and TYK2) consisting of receptor-associated tyrosine kinases that cooperate with seven STAT proteins (STAT1, STAT2, STAT3, STAT4, STAT5A, STAT5B, and STAT6) to regulate cellular functions (Qin et al., 2016). In response to extracellular signals, such as elevated interferon-gamma (IFN-γ) and interleukin-6 (IL-6) levels in PD, JAK is recruited to the cytoplasmic domains of cytokine receptors, activating the pathway (Lashgari et al., 2021). Upon activation, JAK phosphorylates specific tyrosine residues on receptor intracellular domains, creating docking sites for STAT proteins, and phosphorylated STAT proteins dimerize (as homodimers or heterodimers) *via* Src-homology 2 (SH2) domains before translocating to the nucleus to regulate gene expression by binding specific DNA sequences, while feedback regulators such as suppressor of cytokine signaling (SOCS) and protein inhibitors of activated STAT (PIAS) maintain homeostasis by limiting excessive JAK/STAT activation and preventing pathological consequences (Sarapultsev et al., 2023). *In vitro* studies reveal that α-synuclein overexpression enhances STAT3 activation in the SNpc, promoting pro-inflammatory gene expression, including MHC II, iNOS, IL-6, and TNF-α in macrophages and microglia, thereby upregulating the JAK/STAT pathway and fostering a neuroinflammatory environment (Qin et al., 2016). The Line 61-PFF mouse model, serving as a platform for studying the neuroinflammatory mechanisms of PD, identifies two pro-inflammatory immune cell clusters, MM4 and T3, which significantly decrease following treatment with the JAK1/2 inhibitor AZD1480, further revealing that these clusters are driven by the JAK/STAT signaling pathway, thereby highlighting the pivotal regulatory role of this pathway in the neuroinflammatory progression of PD (Hong et al., 2024). In PD, ROS activation of the JAK/STAT pathway serves as a critical link between oxidative stress and neuroinflammation. This activation occurs independently of new protein synthesis, highlighting ROS as a direct upstream activator of the JAK/STAT pathway, and ROS-induced JAK/STAT signaling amplifies inflammation, leading to neuronal death and creating a feedforward cycle that perpetuates oxidative stress, STAT activation, neuroinflammation, and neurodegeneration (Simon et al., 1998). Although JAK/STAT signaling is a convergent inflammatory pathway across multiple neurodegenerative disorders, its activation in PD is more tightly interwoven with redox imbalance and α-synuclein–triggered innate immune activation, in contrast to its broader roles in peripheral immune polarization and demyelination in diseases such as multiple sclerosis and Alzheimer’s disease (Sarapultsev et al., 2023). Precisely modulating this pathway to attenuate neuroinflammation without disrupting its physiological roles in cellular defense and repair remains an unresolved therapeutic challenge requiring fine-tuned, context-specific intervention strategies.

In recent years, the inhibitory effects of SCFAs on the JAK/STAT pathway have been widely studied and acknowledged (Ying, Wei & An, 2021). Both *in vivo* and *in vitro* studies have demonstrated excessive phosphorylation of JAK2 and STAT3 in MPTP-induced PD mouse models and MPP+-treated PC12 cells, while treatment with SCFAs significantly reduced overactivation of the JAK2/STAT3 pathway, along with notable downregulation of oxidative stress markers and pro-inflammatory cytokines, and the specificity of SCFAs’ effects on the JAK2/STAT3 pathway was further validated by the reversal of their inhibitory effects upon treatment with the JAK2 agonist C-A1, highlighting the critical role of the JAK2/STAT3 axis in mediating the neuroprotective effects of butyrate (Ji et al., 2023). However, the effects of SCFAs are cell-specific, as they can enhance the expression of iNOS and nitric oxide (NO) production in intestinal epithelial cells *via* JAK/STAT signaling, displaying pro-inflammatory properties, which contrasts sharply with their anti-inflammatory effects in other cell types (Stempelj et al., 2007). While SCFAs have the potential to alleviate neuroinflammation in the brain, their pro-inflammatory effects necessitate a nuanced approach when considering their application as therapeutic agents for PD.

### PI3K/Akt signaling pathway

The phosphoinositide 3-kinase (PI3K) family is a central component of this pathway, and PI3K is categorized into three types based on its structure and function; however, research indicates that only Class I PI3Ks exhibit lipid kinase activity in response to growth stimuli, activating protein kinase B (PKB, also known as Akt) (Gupta et al., 2022). Class I PI3K comprises a catalytic subunit (p110) and a regulatory subunit (p85), and under normal conditions, it is activated by upstream molecules such as RTKs or GPCRs through growth factor binding, leading to the conversion of phosphatidylinositol 4,5-bisphosphate (PIP2) into phosphatidylinositol 3,4,5-trisphosphate (PIP3); however, acting as a key signaling molecule, PIP3 recruits and activates downstream effectors such as PDK1 and Akt at the cell membrane, thereby initiating a variety of intracellular biological processes (Long et al., 2021; Gupta & Gaykalova, 2024). Akt, a serine/threonine kinase, inhibits the progressive apoptosis of DA neurons in the substantia nigra by phosphorylating Bcl-2 antagonist and FoxO1, while it also regulates nuclear factor erythroid 2-related factor 2 (Nrf2) to enhance antioxidant enzyme expression, thereby mitigating oxidative stress-induced neuronal damage (Long et al., 2021). Akt also directly phosphorylates glycogen synthase kinase-3β (GSK-3β) at the Ser9 site, inhibiting its activity, while the resulting reduction in GSK-3β activity decreases abnormal phosphorylation of α-synuclein, preventing its aggregation and alleviating PD pathology (Yang et al., 2018). Full activation of Akt requires phosphorylation at Thr308 by PDK1 and at Ser473 by the mTORC2 complex, and once activated, Akt phosphorylates various substrates to regulate multiple cellular processes (Sarbassov et al., 2005). Mammalian target of rapamycin (mTOR) serves as a critical downstream target of Akt, forming the mTORC1 and mTORC2 complexes to regulate cell growth, metabolism, autophagy, and protein synthesis (Gupta et al., 2022). Akt activates mTORC1 by phosphorylating and inhibiting tuberous sclerosis complex 2 (TSC2), and subsequently, mTORC1 phosphorylates S6 kinase 1 (S6K1) and 4E-binding protein 1 (4EBP1), promoting protein synthesis, supporting neuronal survival, and enhancing antioxidant defenses (Wong, 2010). In PD, dysregulation of the PI3K/Akt/mTOR pathway may impair neuronal protein synthesis, compromising the repair and survival of DA neurons (Song, Peng & Zhu, 2021). Moreover, the PI3K/Akt/mTOR pathway serves as a critical negative regulator of autophagy, and inhibition of this process can hinder the clearance of abnormally aggregated α-Syn (Zhang et al., 2021). Therefore, prolonged overactivation of the PI3K/Akt/mTOR pathway may disrupt this essential protective autophagy process, thereby exacerbating PD pathology.

Given the prominent role of the PI3K/Akt signaling pathway in autophagy regulation, researchers have explored its interplay with the Atg5-dependent autophagy pathway, and in NaB-treated mouse neuroendocrine STC-1 cells, Atg5 expression was elevated while the PI3K/Akt/mTOR pathway was significantly suppressed, with this suppression being critical for autophagy activation, while this process not only facilitated the degradation of α-synuclein but also appeared to influence apoptosis and inflammatory responses, offering new insights into potential therapeutic mechanisms for PD (Qiao et al., 2020b). Another study using a rat MCAO model to simulate ischemic brain injury demonstrated that NaB binding to GPR41 activated downstream Gβγ subunits, which in turn attenuated neuronal apoptosis through the PI3K/Akt signaling pathway, providing neuroprotection (Zhou et al., 2021). Additionally, propionate was found to activate the PI3K/Akt signaling pathway, enhancing eNOS phosphorylation and increasing NO production, which reduced oxidative stress-induced damage to DA neurons, alleviated neuronal apoptosis, and subsequently slowed disease progression while improving patient symptoms (Wu et al., 2022). In summary, the potential of SCFAs to regulate the PI3K/Akt signaling pathway in PD warrants further investigation. However, practical application of this strategy must address individual variability and elucidate the complexity of underlying mechanisms to ensure safety and efficacy.

### AMPK signaling pathway

AMP-activated Protein Kinase (AMPK) is a critical regulator of cellular energy balance, forming a heterotrimeric complex consisting of a catalytic α subunit and two regulatory subunits, β and γ (Hardie, Carling & Gamblin, 2011). In the early stages of PD, increased oxidative stress exacerbates mitochondrial damage and triggers neuroinflammation (Picca et al., 2020). Under such pathological conditions, ATP consumption rises, leading to elevated levels of AMP and ADP, which bind to the CBS domain of AMPK’s γ subunit and induce conformational changes that expose the Thr-172 site on the α subunit, enhancing phosphorylation by upstream kinases such as liver kinase B1 (LKB1) and calcium/calmodulin-dependent protein kinase kinase 2 (CaMKK2), thereby activating AMPK (Mihaylova & Shaw, 2011). Activated AMPK plays a pivotal role in restoring mitochondrial function, regulating energy metabolism, and enhancing neuronal survival in PD. Recent studies reveal that AMPK activation of SIRT1 effectively combats oxidative stress, triggers autophagy, and suppresses neuroinflammation, and in conjunction with peroxisome proliferator-activated receptor-γ coactivator 1-α (PGC-1α), AMPK enhances mitochondrial biogenesis and function, reduces ROS production, and mitigates oxidative stress-induced neuronal apoptosis, with these combined mechanisms contributing significantly to long-term neuronal recovery (Fan et al., 2024; Elesawy et al., 2024). Furthermore, AMPK actively promotes mitophagy by directly phosphorylating Unc-51-like autophagy activating kinase 1 (ULK1), initiating autophagosome formation and the degradation of damaged mitochondria (Hardie, 2011). The PINK1/Parkin pathway plays a central role in this process, with recent studies showing that AMPK regulates the localization of PINK1 mRNA to mitochondria, which is essential for local PINK1 protein translation and the initiation of mitophagy, and a complex formed by the outer mitochondrial membrane protein SYNJ2BP and the RNA-binding protein SYNJ2 anchors PINK1 mRNA to mitochondria, while AMPK phosphorylates the PDZ domain of SYNJ2BP, enhancing its interaction with SYNJ2 and ensuring the stable connection of PINK1 mRNA to mitochondria, thereby facilitating efficient PINK1 protein translation on locally damaged mitochondria and initiating mitophagy to address mitochondrial damage (Hees et al., 2024). Researchers demonstrated that cytarabine enhances PINK1/Parkin-mediated mitophagy *via* AMPK activation, thereby improving mitochondrial function, reducing oxidative stress, and providing significant neuroprotection in rotenone-induced PD models (Soper et al., 2008). Moreover, recent studies on the AMPK-mTOR-TFEB axis have firmly linked AMPK to α-synuclein degradation, as AMPK inhibits mTOR to initiate autophagy and enhance the nuclear translocation of transcription factor EB (TFEB), a key regulator of lysosome-associated genes, thereby promoting lysosome biogenesis and reducing α-synuclein expression, a finding validated in A53T α-synuclein PD mouse models (Xu et al., 2022a). This discovery opens new therapeutic avenues for previously undruggable diseases, particularly through targeted protein degradation *via* the AMPK-mTOR-TFEB signaling pathway. However, precisely modulating AMPK signaling to avoid potential side effects remains a key focus for future research.

SCFAs have long been recognized for their potential to improve mitochondrial function and provide neuroprotection through activation of the AMPK pathway, which is significant for mitigating the pathological progression of PD (Tang et al., 2011; Herzig & Shaw, 2018). Cognitive impairment, a frequent no mutant ataxin-3 aggregates, and improved motor function, with the activation of the AMPK pathway being central to these effects, suggesting that SCFAs may similarly benefit PD by enhancing autophagy in diseases with comparable pathological features (Watchon et al., 2024). The protective effects of SCFAs against oxidative stress and mitochondrial dysfunction in IPEC-J2 cells provide compelling evidence for their potential application in PD, as SCFAs activate AMPK to promote mitophagy and alleviate oxidative stress, offering a neuroprotective strategy to slow PD progression. Notably, SCFA-mediated activation of AMPK displays a marked dose-dependent profile, wherein escalating concentrations of SCFAs correlate with increasingly robust AMPK phosphorylation, culminating in amplified anti-inflammatory effect outcomes (Li et al., 2023a). These findings highlight the importance of SCFAs and the AMPK pathway in neurodegenerative disease research, paving the way for novel interventions targeting mitochondrial and cellular health in PD (Li et al., 2022). However, current research has primarily focused on metabolic disease models. Further investigation is needed to elucidate the precise mechanisms and long-term effects of SCFA-mediated AMPK activation in PD.

### Nrf2/Keap1/ARE signaling pathway

Nrf2 is a transcription factor that orchestrates cellular antioxidant responses by regulating the expression of various antioxidant and cytoprotective genes, and structurally, Nrf2 contains several highly conserved domains, with the Neh1 domain enabling Nrf2 to form heterodimers with small Maf proteins (MafG, MafK, and MafF) in the nucleus, a crucial step for binding to antioxidant response elements (ARE) in the promoter regions of target genes (Bellezza et al., 2018). The Neh2 domain plays a critical role in Nrf2 regulation by mediating its interaction with Kelch-like ECH-associated protein 1 (Keap1), a 625-amino acid polypeptide also known as cytoskeleton-associated protein 1, which anchors Nrf2 in the cytoplasm and acts as a molecular switch to control its activity (Bryan et al., 2013). Under normal conditions, Nrf2 binds to Keap1 in the cytoplasm to form an inactive complex, with Keap1 targeting Nrf2 for ubiquitination and proteasomal degradation, thereby maintaining Nrf2 at low basal levels and minimizing the transcription of antioxidant genes (Cores et al., 2020). In the progression of PD, oxidative stress and electrophilic compounds modify cysteine residues in the IVR region of Keap1, allowing Nrf2 to evade proteasomal degradation, translocate into the nucleus, form heterodimers with small Maf proteins, and bind to ARE sequences, which activates the transcription of antioxidant enzymes such as γ-glutamylcysteine synthetase (γ-GCS), quinone oxidoreductase 1 (NQO1), and heme oxygenase-1 (HO-1) (Cuadrado et al., 2019).

The activated Nrf2/Keap1/ARE signaling pathway theoretically reduces oxidative stress-induced neuronal damage (Shirgadwar et al., 2023; Shah et al., 2024). However, dysregulation of Nrf2 pathways, particularly interactions with factors like p38, JNK, and Bach1, can lead to insufficient antioxidant gene expression, thereby failing to effectively mitigate oxidative neuronal injury (George et al., 2022). Nevertheless, Nrf2 enhances mitochondrial autophagy by regulating the expression of genes such as PGC-1α (PPAR-γ coactivator-1α), Nrf1, and PINK1, which helps eliminate damaged mitochondria and effectively curtails the production of ROS (Gumeni et al., 2021). Furthermore, experiments with SQSTM1 (p62) plasmid transfection showed that p62 overexpression significantly inhibited ferroptosis in DA neurons by activating the Nrf2/Keap1/ARE pathway, which also regulated mitochondrial autophagy and suppressed ROS generated by iron overload, further demonstrating Nrf2’s key role in maintaining mitochondrial function and alleviating oxidative stress (Yao et al., 2024). Additional studies have shown that Nrf2 regulates the expression of 20S and 26S proteasomes as well as HO-1, facilitating the degradation of α-synuclein aggregates, shortening its half-life, and promoting its clearance, which helps reduce α-synuclein neurotoxicity and slow PD progression (He et al., 2013; Skibinski et al., 2017; Chakkittukandiyil et al., 2022). In inflammatory conditions, Nrf2, stabilized by DJ-1 protein, also plays a significant role in suppressing neuroinflammation (Clements et al., 2006). Nrf2 regulates downstream HO-1 expression, which breaks down heme to produce carbon monoxide (CO) and other byproducts that inhibit NF-κB signaling, and this also activates the anti-inflammatory cytokine IL-10, further mitigating inflammation in PD (Loboda et al., 2016; Ahmed et al., 2017). Moreover, Nrf2 not only reduces the expression and release of pro-inflammatory cytokines, preventing excessive microglial activation, but also effectively suppresses the overactivation of the NLRP3 inflammasome, thereby mitigating the resulting neuroinflammation and ultimately slowing the progression of PD (Rajan et al., 2023; Kaur et al., 2024). Therefore, it is evident that the Nrf2/Keap1/ARE signaling pathway provides multi-faceted protection in PD.

Both *in vitro* and *in vivo* studies have demonstrated that butyrate enhances Nrf2 antioxidant activity by inducing its nuclear translocation through HDAC inhibition, and in Nrf2-knockout mouse models, butyrate activates Nrf2 *via* HDAC inhibition mediated by P300, highlighting butyrate’s critical role in regulating Nrf2 through epigenetic mechanisms in the absence of Nrf2 and suggesting its potential therapeutic value in mitigating oxidative stress and inflammation in PD (Wu et al., 2018). Additionally, evidence from bovine mammary epithelial cells indicates that butyrate promotes Nrf2 nuclear accumulation by activating the GPR109A receptor, which deactivates AMPK signaling, a regulatory mechanism that underscores the diverse pathways through which butyrate modulates Nrf2 activity (Guo et al., 2020). In contrast, fewer studies have investigated propionate. However, evidence from BBB integrity models suggests that propionate promotes Nrf2 nuclear translocation *via* pathways such as GPR41 activation, while concurrently enhancing the expression of tight junction proteins, maintaining BBB integrity, and reducing neuroinflammation, highlighting the neuroprotective potential of propionate in modulating oxidative stress and preserving BBB function (Fock & Parnova, 2023).

## Crosstalk among signaling pathways

Crosstalk between signaling pathways cellular signaling pathways do not function in isolation; rather, they integrate biological signals from various sources through intricate interactions. Dysregulation in one pathway can disrupt its own functionality and trigger cascading effects on other pathways. This crosstalk underlines the complexity of neurodegenerative disease progression, particularly in PD. The intricate network of signaling interactions provides multiple potential therapeutic targets for PD. By modulating these interactions, it may be possible to restore intracellular homeostasis and effectively mitigate neuronal damage. Studies have shown that the effects of PI3K/mTOR inhibitors can be reversed by MEK or ERK inhibition, suggesting a conditional interdependence between the PI3K/Akt and MAPK pathways (Toulany et al., 2014). This crosstalk plays a critical role in regulating neuronal death. Balancing these pathways may offer promising therapeutic approaches for PD. The PI3K/AKT signaling pathway is pivotal for cell survival and anti-apoptotic processes, primarily through NF-κB activation, which enhances the transcriptional activity of p65/RelA, the major subunit of NF-κB, inhibiting apoptosis, and further research has shown that PI3K/AKT signaling activates transcription factors like cAMP response element-binding protein (CREB) or IKK, facilitating downstream NF-κB signaling and amplifying its anti-apoptotic effects (Zhang et al., 2018; Usman et al., 2019). JAK2 kinase has been shown to activate both the PI3K/mTOR and MAPK pathways, and under PI3K/mTOR inhibition, cells employ feedback mechanisms to activate the JAK2/STAT5 pathway, which in turn reactivates the PI3K pathway, with this biphasic activation counteracting PI3K/mTOR inhibition, reinitiating survival pathways during treatment, enhancing cell survival, and potentially increasing invasiveness, contributing to a drug resistance mechanism (Britschgi et al., 2012). Within the Ras/Raf/MAPK/ERK pathway, JAK2 exerts its effects *via* hub proteins, including SH2 domain-containing adapter proteins (SHC), growth factor receptor-bound protein (GRB), and SOS, and activation of these proteins facilitates cellular responsiveness to external signals (Bousoik & Montazeri Aliabadi, 2018). The JAK/STAT pathway is a critical axis for inflammatory signal transmission and plays a central role in amplifying inflammatory feedback loops, and by coordinating with NF-κB, a classic transcription factor, it significantly enhances the expression of pro-inflammatory genes, driving sustained inflammatory responses in PD (Panda et al., 2024). The p65 subunit of NF-κB competes with Nrf2 for binding to CREB-binding protein (CBP), thereby limiting Nrf2-mediated antioxidant gene expression and exacerbating inflammatory responses, while also promoting the nuclear translocation of Keap1 to accelerate Nrf2 degradation and modulating inflammatory factor levels to regulate Nrf2 activity (Cores et al., 2020). Understanding the interaction between NF-κB and Nrf2 not only provides insights into PD pathogenesis but also offers potential avenues for developing novel anti-inflammatory and antioxidant therapies. Extensive research has demonstrated that Nrf2 activation is regulated by AMPK, which directly phosphorylates Nrf2 or its regulatory factors, promoting its nuclear translocation, and their synergistic effects enhance antioxidant capacity, suppress neuroinflammation, and promote autophagy, collectively providing multifaceted protection to neuronal cells (Yasuda et al., 2024). These findings reveal that pathway dysregulation disrupts multiple cellular physiological functions through cascading effects. Restoring balance among these pathways could potentially reestablish dynamic cellular homeostasis and reduce neuronal damage. However, while this complex signaling network offers new therapeutic directions for PD, the high complexity of crosstalk and the unclear roles of specific pathways under varying pathological conditions present challenges. Further research is needed to assess the feasibility and efficacy of specific interventions in clinical settings.

## Conclusions

This study highlights the regulatory potential of SCFAs in PD, positioning phosphorylation signaling as a central conduit through which SCFAs influence α-synuclein aggregation, mitochondrial dysfunction, oxidative stress, and neuroinflammatory responses. Despite substantial progress in mapping these interactions, the precise regulatory logic remains unresolved, owing to extensive crosstalk among MAPKs, NF-κB, JAK/STAT, PI3K/Akt, AMPK, and Nrf2/Keap1/ARE pathways and the highly context-dependent nature of SCFA activity. Current evidence—derived largely from preclinical models—does not yet capture the dynamic, concentration- and stage-specific responses observed *in vivo*, and the scarcity of large-scale clinical studies measuring physiologically relevant SCFA levels continues to obscure their true translational potential. As the field advances, a major challenge lies in defining the molecular specificity of SCFA-responsive phosphorylation events in human PD, disentangling temporal regulatory patterns across disease progression, and accounting for interindividual variability shaped by gut microbiota composition and metabolic output. The integration of multi-omics platforms, including single-cell phosphoproteomics, spatial transcriptomics, and gut–brain metabolomics, will be essential for elucidating SCFAs’ mechanistic roles and identifying actionable biomarkers. Collectively, deepening our understanding of how SCFAs interface with these interconnected signaling networks may not only clarify their contribution to PD pathophysiology but also accelerate the development of targeted, microbiota-informed therapeutic strategies for neurodegenerative disease.

## Additional Information and Declarations

### Data Availability

The following information was supplied regarding data availability:

This is a Literature Review.

## References

[ref-1] Ahmed SMU, Luo L, Namani A, Wang XJ, Tang X (2017). Nrf2 signaling pathway: pivotal roles in inflammation. Biochimica et Biophysica Acta (BBA)—Molecular Basis of Disease.

[ref-2] Aho VTE, Houser MC, Pereira PAB, Chang J, Rudi K, Paulin L, Hertzberg V, Auvinen P, Tansey MG, Scheperjans F (2021). Relationships of gut microbiota, short-chain fatty acids, inflammation, and the gut barrier in Parkinson’s disease. Molecular Neurodegeneration.

[ref-3] Alam P, Bousset L, Melki R, Otzen DE (2019). α-synuclein oligomers and fibrils: a spectrum of species, a spectrum of toxicities. Journal of Neurochemistry.

[ref-4] Anderson JP, Walker DE, Goldstein JM, de Laat R, Banducci K, Caccavello RJ, Barbour R, Huang J, Kling K, Lee M, Diep L, Keim PS, Shen X, Chataway T, Schlossmacher MG, Seubert P, Schenk D, Sinha S, Gai WP, Chilcote TJ (2006). Phosphorylation of ser-129 is the dominant pathological modification of alpha-synuclein in familial and sporadic lewy body disease. Journal of Biological Chemistry.

[ref-5] Ardito F, Giuliani M, Perrone D, Troiano G, Lo Muzio L (2017). The crucial role of protein phosphorylation in cell signaling and its use as targeted therapy (review). International Journal of Molecular Medicine.

[ref-6] Bayazid AB, Jang YA, Kim YM, Kim JG, Lim BO (2021). Neuroprotective effects of sodium butyrate through suppressing neuroinflammation and modulating antioxidant enzymes. Neurochemical Research.

[ref-7] Bellezza I, Giambanco I, Minelli A, Donato R (2018). Nrf2-Keap1 signaling in oxidative and reductive stress. Biochimica et Biophysica Acta (BBA)—Molecular Cell Research.

[ref-8] Bloem BR, Okun MS, Klein C (2021). Parkinson’s disease. Lancet.

[ref-9] Bousoik E, Montazeri Aliabadi H (2018). “Do We Know Jack” about JAK? A closer look at JAK/STAT signaling pathway. Frontiers in Oncology.

[ref-10] Braak H, Del Tredici K, Rüb U, de Vos RAI, Jansen Steur ENH, Braak E (2003a). Staging of brain pathology related to sporadic Parkinson’s disease. Neurobiology of Aging.

[ref-11] Braak H, Rüb U, Gai WP, Del Tredici K (2003b). Idiopathic Parkinson’s disease: possible routes by which vulnerable neuronal types may be subject to neuroinvasion by an unknown pathogen. Journal of Neural Transmission.

[ref-12] Brash-Arias D, García LI, Pérez-Estudillo CA, Rojas-Durán F, Aranda-Abreu GE, Herrera-Covarrubias D, Chi-Castañeda D (2024). The role of astrocytes and alpha-synuclein in Parkinson’s disease: a review. NeuroSci.

[ref-13] Britschgi A, Andraos R, Brinkhaus H, Klebba I, Romanet V, Müller U, Murakami M, Radimerski T, Bentires-Alj M (2012). JAK2/STAT5 inhibition circumvents resistance to PI3K/mTOR blockade: a rationale for cotargeting these pathways in metastatic breast cancer. Cancer Cell.

[ref-14] Brown AJ, Goldsworthy SM, Barnes AA, Eilert MM, Tcheang L, Daniels D, Muir AI, Wigglesworth MJ, Kinghorn I, Fraser NJ, Pike NB, Strum JC, Steplewski KM, Murdock PR, Holder JC, Marshall FH, Szekeres PG, Wilson S, Ignar DM, Foord SM, Wise A, Dowell SJ (2003). The orphan G protein-coupled receptors GPR41 and GPR43 are activated by propionate and other short chain carboxylic acids. Journal of Biological Chemistry.

[ref-15] Bryan HK, Olayanju A, Goldring CE, Park BK (2013). The Nrf2 cell defence pathway: Keap1-dependent and -independent mechanisms of regulation. Biochemical Pharmacology.

[ref-16] Caetano-Silva ME, Rund L, Hutchinson NT, Woods JA, Steelman AJ, Johnson RW (2023). Inhibition of inflammatory microglia by dietary fiber and short-chain fatty acids. Scientific Reports.

[ref-17] Caetano-Silva ME, Rund L, Vailati-Riboni M, Matt S, Soto-Diaz K, Beever J, Allen JM, Woods JA, Steelman AJ, Johnson RW (2024). The emergence of inflammatory microglia during gut inflammation is not affected by FFAR2 expression in intestinal epithelial cells or peripheral myeloid cells. Brain, Behavior and Immunity.

[ref-18] Chai JT, Digby JE, Choudhury RP (2013). GPR109A and vascular inflammation. Current Atherosclerosis Reports.

[ref-19] Chakkittukandiyil A, Sajini DV, Karuppaiah A, Selvaraj D (2022). The principal molecular mechanisms behind the activation of Keap1/Nrf2/ARE pathway leading to neuroprotective action in Parkinson’s disease. Neurochemistry International.

[ref-20] Chauhan AK, Mittra N, Kumar V, Patel DK, Singh C (2016). Inflammation and B-cell lymphoma-2 associated X protein regulate zinc-induced apoptotic degeneration of rat nigrostriatal dopaminergic neurons. Molecular Neurobiology.

[ref-21] Chen S-J, Chen C-C, Liao H-Y, Lin Y-T, Wu Y-W, Liou J-M, Wu M-S, Kuo C-H, Lin C-H (2022). Association of fecal and plasma levels of short-chain fatty acids with gut microbiota and clinical severity in patients with Parkinson disease. Neurology.

[ref-22] Chen L, Feany MB (2005). Alpha-synuclein phosphorylation controls neurotoxicity and inclusion formation in a drosophila model of Parkinson disease. Nature Neuroscience.

[ref-23] Chen J, Ren Y, Gui C, Zhao M, Wu X, Mao K, Li W, Zou F (2018). Phosphorylation of parkin at serine 131 by p38 MAPK promotes mitochondrial dysfunction and neuronal death in mutant A53T α-synuclein model of Parkinson’s disease. Cell Death and Disease.

[ref-24] Chen PS, Wang C-C, Bortner CD, Peng G-S, Wu X, Pang H, Lu R-B, Gean P-W, Chuang D-M, Hong J-S (2007). Valproic acid and other histone deacetylase inhibitors induce microglial apoptosis and attenuate lipopolysaccharide-induced dopaminergic neurotoxicity. Neuroscience.

[ref-25] Christiansen CB, Gabe MBN, Svendsen B, Dragsted LO, Rosenkilde MM, Holst JJ (2018). The impact of short-chain fatty acids on GLP-1 and PYY secretion from the isolated perfused rat colon. American Journal of Physiology-Gastrointestinal and Liver Physiology.

[ref-26] Cildir G, Low KC, Tergaonkar V (2016). Noncanonical NF-κB signaling in health and disease. Trends in Molecular Medicine.

[ref-27] Clements CM, McNally RS, Conti BJ, Mak TW, Ting JP-Y (2006). DJ-1, a cancer- and Parkinson’s disease-associated protein, stabilizes the antioxidant transcriptional master regulator Nrf2. Proceedings of the National Academy of Sciences of the United States of America.

[ref-28] Contaldi E, Magistrelli L, Comi C (2022). T lymphocytes in Parkinson’s disease. Journal of Parkinson’s Disease.

[ref-29] Cores Á, Piquero M, Villacampa M, León R, Menéndez JC (2020). NRF2 regulation processes as a source of potential drug targets against neurodegenerative diseases. Biomolecules.

[ref-30] Coso OA, Chiariello M, Yu JC, Teramoto H, Crespo P, Xu N, Miki T, Gutkind JS (1995). The small GTP-binding proteins Rac1 and Cdc42 regulate the activity of the JNK/SAPK signaling pathway. Cell.

[ref-31] Cuadrado A, Rojo AI, Wells G, Hayes JD, Cousin SP, Rumsey WL, Attucks OC, Franklin S, Levonen A-L, Kensler TW, Dinkova-Kostova AT (2019). Therapeutic targeting of the NRF2 and KEAP1 partnership in chronic diseases. Nature Reviews Drug Discovery.

[ref-32] Cummings JH, Pomare EW, Branch WJ, Naylor CP, Macfarlane GT (1987). Short chain fatty acids in human large intestine, portal, hepatic and venous blood. Gut.

[ref-33] Ding Y, Xin C, Zhang C-W, Lim K-L, Zhang H, Fu Z, Li L, Huang W (2018). Natural molecules from Chinese herbs protecting against Parkinson’s disease via anti-oxidative stress. Frontiers in Aging Neuroscience.

[ref-34] Dolatshahi M, Ranjbar Hameghavandi MH, Sabahi M, Rostamkhani S (2021). Nuclear factor-kappa B (NF-κB) in pathophysiology of Parkinson disease: diverse patterns and mechanisms contributing to neurodegeneration. European Journal of Neuroscience.

[ref-35] Dorsey ER, Sherer T, Okun MS, Bloem BR (2018). The emerging evidence of the Parkinson pandemic. Journal of Parkinson’s Disease.

[ref-36] Du Y, He C, An Y, Huang Y, Zhang H, Fu W, Wang M, Shan Z, Xie J, Yang Y, Zhao B (2024). The role of short chain fatty acids in inflammation and body health. International Journal of Molecular Sciences.

[ref-37] Duan W-X, Wang F, Liu J-Y, Liu C-F (2023). Relationship between short-chain fatty acids and Parkinson’s disease: a review from pathology to clinic. Neuroscience Bulletin.

[ref-38] Elesawy WH, El-Sahar AE, Sayed RH, Ashour AM, Alsufyani SE, Arab HH, Kandil EA (2024). Repurposing ezetimibe as a neuroprotective agent in a rotenone-induced Parkinson’s disease model in rats: role of AMPK/SIRT-1/PGC-1α signaling and autophagy. International Immunopharmacology.

[ref-39] Espinosa-Oliva AM, Ruiz R, Soto MS, Boza-Serrano A, Rodriguez-Perez AI, Roca-Ceballos MA, García-Revilla J, Santiago M, Serres S, Economopoulus V, Carvajal AE, Vázquez-Carretero MD, García-Miranda P, Klementieva O, Oliva-Martín MJ, Deierborg T, Rivas E, Sibson NR, Labandeira-García JL, Machado A, Peral MJ, Herrera AJ, Venero JL, de Pablos RM (2024). Inflammatory bowel disease induces pathological α-synuclein aggregation in the human gut and brain. Neuropathology and Applied Neurobiology.

[ref-40] Fan J-J, Ding W-D, Liang Y-F, Wei Y-X, Huang Y, Ma L, Wang R (2024). Diosgenin derivative ML5 attenuates MPTP-induced neuronal impairment via regulating AMPK/PGC-1α-mediated mitochondrial biogenesis and fusion/fission. American Journal of Translational Research.

[ref-41] Fang C, Zuo K, Liu Z, Xu L, Yang X (2024). Disordered GPR43/NLRP3 expression in peripheral leukocytes of patients with atrial fibrillation is associated with intestinal short chain fatty acids levels. European Journal of Medical Research.

[ref-42] Fock E, Parnova R (2023). Mechanisms of blood-brain barrier protection by microbiota-derived short-chain fatty acids. Cells.

[ref-43] GBD 2016 Neurology Collaborators (2019). Neurology collaborators, global, regional, and national burden of neurological disorders, 1990–2016: a systematic analysis for the global burden of disease study 2016. The Lancet Neurology.

[ref-44] George M, Tharakan M, Culberson J, Reddy AP, Reddy PH (2022). Role of Nrf2 in aging, Alzheimer’s and other neurodegenerative diseases. Ageing Research Reviews.

[ref-45] Getachew B, Csoka AB, Bhatti A, Copeland RL, Tizabi Y (2020). Butyrate protects against salsolinol-induced toxicity in SH-SY5Y cells: implication for Parkinson’s disease. Neurotoxicity Research.

[ref-46] Gil-Martinez AL, Cuenca-Bermejo L, Gallo-Soljancic P, Sanchez-Rodrigo C, Izura V, Steinbusch HWM, Fernandez-Villalba E, Herrero MT (2020). Study of the link between neuronal death, glial response, and MAPK pathway in old parkinsonian mice. Frontiers in Aging Neuroscience.

[ref-47] Gill RK, Saksena S, Alrefai WA, Sarwar Z, Goldstein JL, Carroll RE, Ramaswamy K, Dudeja PK (2005). Expression and membrane localization of MCT isoforms along the length of the human intestine. American Journal of Physiology-Cell Physiology.

[ref-48] Gitler AD, Bevis BJ, Shorter J, Strathearn KE, Hamamichi S, Su LJ, Caldwell KA, Caldwell GA, Rochet J-C, McCaffery JM, Barlowe C, Lindquist S (2007). The Parkinson’s disease protein α-synuclein disrupts cellular Rab homeostasis. Proceedings of the National Academy of Sciences of the United States of America.

[ref-49] Gray GK, McFarland BC, Nozell SE, Benveniste EN (2014). NF-κB and STAT3 in glioblastoma: therapeutic targets coming of age. Expert Review of Neurotherapeutics.

[ref-50] Gumeni S, Papanagnou E-D, Manola MS, Trougakos IP (2021). Nrf2 activation induces mitophagy and reverses parkin/Pink1 knock down-mediated neuronal and muscle degeneration phenotypes. Cell Death and Disease.

[ref-51] Guo J, Ke S, Chen Q, Zhou J, Guo J, Qiu T (2023a). NCOA7 regulates growth and metastasis of clear cell renal cell carcinoma via MAPK/ERK signaling pathway. International Journal of Molecular Sciences.

[ref-52] Guo W, Liu J, Sun J, Gong Q, Ma H, Kan X, Cao Y, Wang J, Fu S (2020). Butyrate alleviates oxidative stress by regulating NRF2 nuclear accumulation and H3K9/14 acetylation via GPR109A in bovine mammary epithelial cells and mammary glands. Free Radical Biology & Medicine.

[ref-53] Guo B, Zhang J, Zhang W, Chen F, Liu B (2023b). Gut microbiota-derived short chain fatty acids act as mediators of the gut-brain axis targeting age-related neurodegenerative disorders: a narrative review. Critical Reviews in Food Science and Nutrition.

[ref-54] Gupta I, Gaykalova DA (2024). Unveiling the role of PIK3R1 in cancer: a comprehensive review of regulatory signaling and therapeutic implications. Seminars in Cancer Biology.

[ref-55] Gupta S, Kumar M, Chaudhuri S, Kumar A (2022). The non-canonical nuclear functions of key players of the PI3K-AKT-MTOR pathway. Journal of Cellular Physiology.

[ref-56] Gurung P, Lukens JR, Kanneganti T-D (2015). Mitochondria: diversity in the regulation of the NLRP3 inflammasome. Trends in Molecular Medicine.

[ref-57] Hardie DG (2011). AMPK and autophagy get connected. EMBO Journal.

[ref-58] Hardie DG, Carling D, Gamblin SJ (2011). AMP-activated protein kinase: also regulated by ADP?. Trends in Biochemical Sciences.

[ref-59] Hays KE, Pfaffinger JM, Ryznar R (2024). The interplay between gut microbiota, short-chain fatty acids, and implications for host health and disease. Gut Microbes.

[ref-60] He Q, Song N, Jia F, Xu H, Yu X, Xie J, Jiang H (2013). Role of α-synuclein aggregation and the nuclear factor E2-related factor 2/heme oxygenase-1 pathway in iron-induced neurotoxicity. International Journal of Biochemistry & Cell Biology.

[ref-61] Hees JT, Wanderoy S, Lindner J, Helms M, Murali Mahadevan H, Harbauer AB (2024). Insulin signalling regulates Pink1 mRNA localization via modulation of AMPK activity to support PINK1 function in neurons. Nature Metabolism.

[ref-62] Herzig S, Shaw RJ (2018). AMPK: guardian of metabolism and mitochondrial homeostasis. Nature Reviews Molecular Cell Biology.

[ref-63] Heyninck K, Beyaert R (2001). Crosstalk between NF-kappaB-activating and apoptosis-inducing proteins of the TNF-receptor complex. Molecular Cell Biology Research Communications: MCBRC.

[ref-64] Hong H, Wang Y, Menard M, Buckley JA, Zhou L, Volpicelli-Daley L, Standaert DG, Qin H, Benveniste EN (2024). Suppression of the JAK/STAT pathway inhibits neuroinflammation in the line 61-PFF mouse model of Parkinson’s disease. Journal of Neuroinflammation.

[ref-65] Hou Y, Li X, Liu C, Zhang M, Zhang X, Ge S, Zhao L (2021a). Neuroprotective effects of short-chain fatty acids in MPTP induced mice model of Parkinson’s disease. Experimental Gerontology.

[ref-66] Hou Y-F, Shan C, Zhuang S-Y, Zhuang Q-Q, Ghosh A, Zhu K-C, Kong X-K, Wang S-M, Gong Y-L, Yang Y-Y, Tao B, Sun L-H, Zhao H-Y, Guo X-Z, Wang W-Q, Ning G, Gu Y-Y, Li S-T, Liu J-M (2021b). Gut microbiota-derived propionate mediates the neuroprotective effect of osteocalcin in a mouse model of Parkinson’s disease. Microbiome.

[ref-67] Hu C, Zeng D, Huang Y, Deng Q, Liu S, Zhou W, Zhou W (2024). Sodium butyrate ameliorates atopic dermatitis-induced inflammation by inhibiting HDAC3-mediated STAT1 and NF-κB pathway. Inflammation.

[ref-68] Huang W, Man Y, Gao C, Zhou L, Gu J, Xu H, Wan Q, Long Y, Chai L, Xu Y, Xu Y (2020). Short-chain fatty acids ameliorate diabetic nephropathy via GPR43-mediated inhibition of oxidative stress and NF-κB signaling. Oxidative Medicine and Cellular Longevity.

[ref-69] Hwang CJ, Kim YE, Son DJ, Park MH, Choi D-Y, Park P-H, Hellström M, Han S-B, Oh K-W, Park EK, Hong JT (2016). Parkin deficiency exacerbate ethanol-induced dopaminergic neurodegeneration by P38 pathway dependent inhibition of autophagy and mitochondrial function. Redox Biology.

[ref-70] Inoue D, Tsujimoto G, Kimura I (2014). Regulation of energy homeostasis by GPR41. Frontiers in Endocrinology.

[ref-71] Ji L-L, Huang T-T, Mao L-L, Xu Y-F, Chen W-Y, Wang W-W, Wang L-H (2023). The gut microbiota metabolite butyrate mitigates MPTP/MPP+—induced Parkinson’s disease by inhibiting the JAK2/STAT3 signaling pathway. Kaohsiung Journal of Medical Sciences.

[ref-72] Jin X, Dong W, Chang K, Yan Y (2024). Research on the signaling pathways related to the intervention of traditional Chinese medicine in Parkinson’s disease: a literature review. Journal of Ethnopharmacology.

[ref-73] Jin H, Kanthasamy A, Harischandra DS, Kondru N, Ghosh A, Panicker N, Anantharam V, Rana A, Kanthasamy AG (2014). Histone hyperacetylation up-regulates protein kinase Cδ in dopaminergic neurons to induce cell death: relevance to epigenetic mechanisms of neurodegeneration in Parkinson disease. Journal of Biological Chemistry.

[ref-74] Kaji I, Iwanaga T, Watanabe M, Guth PH, Engel E, Kaunitz JD, Akiba Y (2015). SCFA transport in rat duodenum. American Journal of Physiology-Gastrointestinal and Liver Physiology.

[ref-75] Kakoty V, Sarathlal KC, Dubey SK, Yang CH, Kesharwani P, Taliyan R (2021a). The gut-brain connection in the pathogenicity of Parkinson disease: putative role of autophagy. Neuroscience Letters.

[ref-76] Kakoty V, Sarathlal KC, Dubey SK, Yang C-H, Taliyan R (2021b). Neuroprotective effects of trehalose and sodium butyrate on preformed fibrillar form of α-synuclein-induced rat model of Parkinson’s disease. ACS Chemical Neuroscience.

[ref-77] Kasubuchi M, Hasegawa S, Hiramatsu T, Ichimura A, Kimura I (2015). Dietary gut microbial metabolites, short-chain fatty acids, and host metabolic regulation. Nutrients.

[ref-78] Kaur T, Sidana P, Kaur N, Choubey V, Kaasik A (2024). Unraveling neuroprotection in Parkinson’s disease: Nrf2-Keap1 pathway’s vital role amidst pathogenic pathways. Inflammopharmacology.

[ref-79] Khot M, Sood A, Tryphena KP, Khan S, Srivastava S, Singh SB, Khatri DK (2022). NLRP3 inflammasomes: a potential target to improve mitochondrial biogenesis in Parkinson’s disease. European Journal of Pharmacology.

[ref-80] Kim MH, Kang SG, Park JH, Yanagisawa M, Kim CH (2013). Short-chain fatty acids activate GPR41 and GPR43 on intestinal epithelial cells to promote inflammatory responses in mice. Gastroenterology.

[ref-81] Kimura I, Inoue D, Maeda T, Hara T, Ichimura A, Miyauchi S, Kobayashi M, Hirasawa A, Tsujimoto G (2011). Short-chain fatty acids and ketones directly regulate sympathetic nervous system via G protein-coupled receptor 41 (GPR41). Proceedings of the National Academy of Sciences of the United States of America.

[ref-82] Koh A, De Vadder F, Kovatcheva-Datchary P, Bäckhed F (2016). From dietary fiber to host physiology: short-chain fatty acids as key bacterial metabolites. Cell.

[ref-83] Kordower JH, Chu Y, Hauser RA, Freeman TB, Olanow CW (2008). Lewy body-like pathology in long-term embryonic nigral transplants in Parkinson’s disease. Nature Medicine.

[ref-84] Krzystek TJ, Banerjee R, Thurston L, Huang J, Swinter K, Rahman SN, Falzone TL, Gunawardena S (2021). Differential mitochondrial roles for α-synuclein in DRP1-dependent fission and PINK1/parkin-mediated oxidation. Cell Death and Disease.

[ref-85] Kumar D, Bishnoi M, Kondepudi KK, Sharma SS (2025). Gut microbiota-based interventions for Parkinson’s disease: neuroprotective mechanisms and current perspective. Probiotics and Antimicrobial Proteins.

[ref-86] Lashgari N-A, Roudsari NM, Momtaz S, Sathyapalan T, Abdolghaffari AH, Sahebkar A (2021). The involvement of JAK/STAT signaling pathway in the treatment of Parkinson’s disease. Journal of Neuroimmunology.

[ref-87] Layden BT, Angueira AR, Brodsky M, Durai V, Lowe WL (2013). Short chain fatty acids and their receptors: new metabolic targets. Translational Research: the Journal of Laboratory and Clinical Medicine.

[ref-88] Leandrou E, Chalatsa I, Anagnostou D, Machalia C, Semitekolou M, Filippa V, Makridakis M, Vlahou A, Anastasiadou E, Vekrellis K, Emmanouilidou E (2024). α-synuclein oligomers potentiate neuroinflammatory NF-κB activity and induce Cav3.2 calcium signaling in astrocytes. Translational Neurodegeneration.

[ref-89] Lee AK, Kim DH, Bang E, Choi YJ, Chung HY (2020). β-hydroxybutyrate suppresses lipid accumulation in aged liver through GPR109A-mediated signaling. Aging and Disease.

[ref-90] Lehtonen Š, Sonninen T-M, Wojciechowski S, Goldsteins G, Koistinaho J (2019). Dysfunction of cellular proteostasis in Parkinson’s disease. Frontiers in Neuroscience.

[ref-91] Leite JA, Ghirotto B, Targhetta VP, de Lima J, Câmara NOS (2022). Sirtuins as pharmacological targets in neurodegenerative and neuropsychiatric disorders. British Journal of Pharmacology.

[ref-92] Li X, Chen L-M, Kumar G, Zhang S-J, Zhong Q-H, Zhang H-Y, Gui G, Wu L-L, Fan H-Z, Sheng J-W (2022). Therapeutic interventions of gut-brain axis as novel strategies for treatment of alcohol use disorder associated cognitive and mood dysfunction. Frontiers in Neuroscience.

[ref-93] Li W, Deng M, Gong J, Hou Y, Zhao L (2023a). Bidirectional regulation of sodium acetate on macrophage activity and its role in lipid metabolism of hepatocytes. International Journal of Molecular Sciences.

[ref-94] Li J-Y, Englund E, Holton JL, Soulet D, Hagell P, Lees AJ, Lashley T, Quinn NP, Rehncrona S, Björklund A, Widner H, Revesz T, Lindvall O, Brundin P (2008). Lewy bodies in grafted neurons in subjects with Parkinson’s disease suggest host-to-graft disease propagation. Nature Medicine.

[ref-95] Li Q, Li S, Fang J, Yang C, Zhao X, Wang Q, Zhou W, Zheng W (2023b). Artemisinin confers neuroprotection against 6-OHDA-induced neuronal injury in vitro and in vivo through activation of the ERK1/2 pathway. Molecules.

[ref-96] Li M, Tian Y, Wen X, Fu J, Gao J, Zhu Y (2024). Inhibition of thioredoxin reductase and upregulation of apoptosis genes for effective anti-tumor sono-chemotherapy using a meso-organosilica nanomedicine. Biomaterials Science.

[ref-97] Li X, Wang C, Zhu J, Lin Q, Yu M, Wen J, Feng J, Hu C, Wu H (2022). Sodium butyrate ameliorates oxidative stress-induced intestinal epithelium barrier injury and mitochondrial damage through AMPK-mitophagy pathway. Oxidative Medicine and Cellular Longevity.

[ref-98] Liao P-H, Tung H-Y, Lim WS, Jang J-SR, Li H, Shun C-T, Chiu H-M, Wu M-S, Lin C-H (2024). Impaired gut barrier integrity and reduced colonic expression of free fatty acid receptors in patients with Parkinson’s disease. Neurological Sciences: Official Journal of the Italian Neurological Society and of the Italian Society of Clinical Neurophysiology.

[ref-99] Liu Z, Li C, Wu G, Li W, Zhang X, Zhou J, Zhang L, Tao J, Shen M, Liu H (2020). Involvement of JNK/FOXO1 pathway in apoptosis induced by severe hypoxia in porcine granulosa cells. Theriogenology.

[ref-100] Liu J, Shi M, Hong Z, Zhang J, Bradner J, Quinn T, Beyer RP, Mcgeer PL, Chen S, Zhang J (2010). Identification of ciliary neurotrophic factor receptor alpha as a mediator of neurotoxicity induced by alpha-synuclein. Proteomics.

[ref-101] Liu J, Wang F, Liu S, Du J, Hu X, Xiong J, Fang R, Chen W, Sun J (2017). Sodium butyrate exerts protective effect against Parkinson’s disease in mice via stimulation of glucagon like peptide-1. Journal of the Neurological Sciences.

[ref-102] Loboda A, Damulewicz M, Pyza E, Jozkowicz A, Dulak J (2016). Role of Nrf2/HO-1 system in development, oxidative stress response and diseases: an evolutionarily conserved mechanism. Cellular and Molecular Life Sciences.

[ref-103] Long H-Z, Cheng Y, Zhou Z-W, Luo H-Y, Wen D-D, Gao L-C (2021). PI3K/AKT signal pathway: a target of natural products in the prevention and treatment of Alzheimer’s disease and Parkinson’s disease. Frontiers in Pharmacology.

[ref-104] Luk KC, Kehm V, Carroll J, Zhang B, O’Brien P, Trojanowski JQ, Lee VM-Y (2012). Pathological α-synuclein transmission initiates parkinson-like neurodegeneration in nontransgenic mice. Science.

[ref-105] Malpartida AB, Williamson M, Narendra DP, Wade-Martins R, Ryan BJ (2021). Mitochondrial dysfunction and mitophagy in Parkinson’s disease: from mechanism to therapy. Trends in Biochemical Sciences.

[ref-106] Mani S, Sevanan M, Krishnamoorthy A, Sekar S (2021). A systematic review of molecular approaches that link mitochondrial dysfunction and neuroinflammation in Parkinson’s disease. Neurological Sciences: Official Journal of the Italian Neurological Society and of the Italian Society of Clinical Neurophysiology.

[ref-107] Mariani L-L, Longueville S, Girault J-A, Hervé D, Gervasi N (2019). Differential enhancement of ERK, PKA and Ca2+ signaling in direct and indirect striatal neurons of parkinsonian mice. Neurobiology of Disease.

[ref-108] Meesters RJW, van Eijk HMH, ten Have GAM, de Graaf AA, Venema K, van Rossum BEJ, Deutz NEP (2007). Application of liquid chromatography-mass spectrometry to measure the concentrations and study the synthesis of short chain fatty acids following stable isotope infusions. Journal of Chromatography. B, Analytical Technologies in the Biomedical and Life Sciences.

[ref-109] Mehra S, Sahay S, Maji SK (2019). α-synuclein misfolding and aggregation: implications in Parkinson’s disease pathogenesis. Biochimica et Biophysica Acta (BBA)—Proteins and Proteomics.

[ref-110] Mihaylova MM, Shaw RJ (2011). The AMP-activated protein kinase (AMPK) signaling pathway coordinates cell growth, autophagy, & metabolism. Nature Cell Biology.

[ref-111] Mishra T, Singh S, Singh TG (2024). Therapeutic implications and regulations of protein post-translational modifications in Parkinson’s disease. Cellular and Molecular Neurobiology.

[ref-112] Moreau NM, Goupry SM, Antignac JP, Monteau FJ, Le Bizec BJ, Champ MM, Martin LJ, Dumon HJ (2003). Simultaneous measurement of plasma concentrations and 13C-enrichment of short-chain fatty acids, lactic acid and ketone bodies by gas chromatography coupled to mass spectrometry. Journal of Chromatography. B, Analytical Technologies in the Biomedical and Life Sciences.

[ref-113] Morris HR, Spillantini MG, Sue CM, Williams-Gray CH (2024). The pathogenesis of Parkinson’s disease. Lancet.

[ref-114] Mou Y, Du Y, Zhou L, Yue J, Hu X, Liu Y, Chen S, Lin X, Zhang G, Xiao H, Dong B (2022). Gut microbiota interact with the brain through systemic chronic inflammation: implications on neuroinflammation, neurodegeneration, and aging. Frontiers in Immunology.

[ref-115] Munhoz RP, Tumas V, Pedroso JL, Silveira-Moriyama L (2024). The clinical diagnosis of Parkinson’s disease. Arquivos de Neuro-Psiquiatria.

[ref-116] Naren P, Cholkar A, Kamble S, Khan SS, Srivastava S, Madan J, Mehra N, Tiwari V, Singh SB, Khatri DK (2023a). Pathological and therapeutic advances in Parkinson’s disease: mitochondria in the interplay. Journal of Alzheimer’s Disease: JAD.

[ref-117] Naren P, Samim KS, Tryphena KP, Vora LK, Srivastava S, Singh SB, Khatri DK (2023b). Microtubule acetylation dyshomeostasis in Parkinson’s disease. Translational Neurodegeneration.

[ref-118] Narendra DP, Youle RJ (2024). The role of PINK1-parkin in mitochondrial quality control. Nature Cell Biology.

[ref-119] Nicoletti V, Palermo G, Del Prete E, Mancuso M, Ceravolo R (2021). Understanding the multiple role of mitochondria in Parkinson’s disease and related disorders: lesson from genetics and protein-interaction network. Frontiers in Cell and Developmental Biology.

[ref-120] Panda SP, Kesharwani A, Datta S, Prasanth DSNBK, Panda SK, Guru A (2024). JAK2/STAT3 as a new potential target to manage neurodegenerative diseases: an interactive review. European Journal of Pharmacology.

[ref-121] Parra-Rivas LA, Madhivanan K, Aulston BD, Wang L, Prakashchand DD, Boyer NP, Saia-Cereda VM, Branes-Guerrero K, Pizzo DP, Bagchi P, Sundar VS, Tang Y, Das U, Scott DA, Rangamani P, Ogawa Y, Subhojit Roy N (2023). Serine-129 phosphorylation of α-synuclein is an activity-dependent trigger for physiologic protein-protein interactions and synaptic function. Neuron.

[ref-122] Picca A, Calvani R, Coelho-Junior HJ, Landi F, Bernabei R, Marzetti E (2020). Mitochondrial dysfunction, oxidative stress, and neuroinflammation: intertwined roads to neurodegeneration. Antioxidants.

[ref-123] Psichas A, Sleeth ML, Murphy KG, Brooks L, Bewick GA, Hanyaloglu AC, Ghatei MA, Bloom SR, Frost G (2015). The short chain fatty acid propionate stimulates GLP-1 and PYY secretion via free fatty acid receptor 2 in rodents. International Journal of Obesity (2005).

[ref-124] Qiao C-M, Sun M-F, Jia X-B, Li Y, Zhang B-P, Zhao L-P, Shi Y, Zhou Z-L, Zhu Y-L, Cui C, Shen Y-Q (2020a). Sodium butyrate exacerbates Parkinson’s disease by aggravating neuroinflammation and colonic inflammation in MPTP-induced mice model. Neurochemical Research.

[ref-125] Qiao C-M, Sun M-F, Jia X-B, Shi Y, Zhang B-P, Zhou Z-L, Zhao L-P, Cui C, Shen Y-Q (2020b). Sodium butyrate causes α-synuclein degradation by an Atg5-dependent and PI3K/Akt/mTOR-related autophagy pathway. Experimental Cell Research.

[ref-126] Qin H, Buckley JA, Li X, Liu Y, Fox TH, Meares GP, Yu H, Yan Z, Harms AS, Li Y, Standaert DG, Benveniste EN (2016). Inhibition of the JAK/STAT pathway protects against α-synuclein-induced neuroinflammation and dopaminergic neurodegeneration. The Journal of Neuroscience: the Official Journal of the Society for Neuroscience.

[ref-127] Rai SN, Dilnashin H, Birla H, Singh SS, Zahra W, Rathore AS, Singh BK, Singh SP (2019). The role of PI3K/akt and ERK in neurodegenerative disorders. Neurotoxicity Research.

[ref-128] Rajan S, Tryphena KP, Khan S, Vora L, Srivastava S, Singh SB, Khatri DK (2023). Understanding the involvement of innate immunity and the Nrf2-NLRP3 axis on mitochondrial health in Parkinson’s disease. Ageing Research Reviews.

[ref-129] Recasens A, Ulusoy A, Kahle PJ, Di Monte DA, Dehay B (2018). In vivo models of alpha-synuclein transmission and propagation. Cell and Tissue Research.

[ref-130] Ren X, Zou L, Zhang X, Branco V, Wang J, Carvalho C, Holmgren A, Lu J (2017). Redox signaling mediated by thioredoxin and glutathione systems in the central nervous system. Antioxidants & Redox Signaling.

[ref-131] Roediger WE (1980). Role of anaerobic bacteria in the metabolic welfare of the colonic mucosa in man. Gut.

[ref-132] Salles A, Romano A, Freudenthal R (2014). Synaptic NF-kappa B pathway in neuronal plasticity and memory. Journal of Physiology-Paris.

[ref-133] Sampson TR, Debelius JW, Thron T, Janssen S, Shastri GG, Ilhan ZE, Challis C, Schretter CE, Rocha S, Gradinaru V, Chesselet M-F, Keshavarzian A, Shannon KM, Krajmalnik-Brown R, Wittung-Stafshede P, Knight R, Mazmanian SK (2016). Gut microbiota regulate motor deficits and neuroinflammation in a model of Parkinson’s disease. Cell.

[ref-134] Sarapultsev A, Gusev E, Komelkova M, Utepova I, Luo S, Hu D (2023). JAK-STAT signaling in inflammation and stress-related diseases: implications for therapeutic interventions. Molecular Biomedicine.

[ref-135] Sarbassov DD, Guertin DA, Ali SM, Sabatini DM (2005). Phosphorylation and regulation of akt/PKB by the rictor-mTOR complex. Science.

[ref-136] Schweighauser M, Shi Y, Tarutani A, Kametani F, Murzin AG, Ghetti B, Matsubara T, Tomita T, Ando T, Hasegawa K, Murayama S, Yoshida M, Hasegawa M, Scheres SHW, Goedert M (2020). Structures of α-synuclein filaments from multiple system atrophy. Nature.

[ref-137] Shah S, Pushpa Tryphena K, Singh G, Kulkarni A, Pinjala P, Kumar Khatri D (2024). Neuroprotective role of carvacrol via Nrf2/HO-1/NLRP3 axis in rotenone-induced PD mice model. Brain Research.

[ref-138] Sharma K, Chib S, Gupta A, Singh R, Chalotra R (2024). Interplay between α-synuclein and parkin genes: insights of Parkinson’s disease. Molecular Biology Reports.

[ref-139] Shirgadwar SM, Kumar R, Preeti K, Khatri DK, Singh SB (2023). Neuroprotective effect of phloretin in rotenone-induced mice model of Parkinson’s disease: modulating mTOR-NRF2-p62 mediated autophagy-oxidative stress crosstalk. Journal of Alzheimer’s Disease: JAD.

[ref-140] Silva YP, Bernardi A, Frozza RL (2020). The role of short-chain fatty acids from gut microbiota in gut-brain communication. Frontiers in Endocrinology.

[ref-141] Simon AR, Rai U, Fanburg BL, Cochran BH (1998). Activation of the JAK-STAT pathway by reactive oxygen species. American Journal of Physiology.

[ref-142] Singh F, Ganley IG (2021). Parkinson’s disease and mitophagy: an emerging role for LRRK2. Biochemical Society Transactions.

[ref-143] Skibinski G, Hwang V, Ando DM, Daub A, Lee AK, Ravisankar A, Modan S, Finucane MM, Shaby BA, Finkbeiner S (2017). Nrf2 mitigates LRRK2- and α-synuclein-induced neurodegeneration by modulating proteostasis. Proceedings of the National Academy of Sciences of the United States of America.

[ref-144] Slaughter MJ, Shanle EK, Khan A, Chua KF, Hong T, Boxer LD, Allis CD, Josefowicz SZ, Garcia BA, Rothbart SB, Strahl BD, Davis IJ (2021). HDAC inhibition results in widespread alteration of the histone acetylation landscape and BRD4 targeting to gene bodies. Cell Reports.

[ref-145] Song Q, Peng S, Zhu X (2021). Baicalein protects against MPP+/MPTP-induced neurotoxicity by ameliorating oxidative stress in SH-SY5Y cells and mouse model of Parkinson’s disease. Neurotoxicology.

[ref-146] Soper JH, Roy S, Stieber A, Lee E, Wilson RB, Trojanowski JQ, Burd CG, Lee VM-Y (2008). Alpha-synuclein-induced aggregation of cytoplasmic vesicles in saccharomyces cerevisiae. Molecular Biology of the Cell.

[ref-147] Soraci L, Gambuzza ME, Biscetti L, Laganà P, Lo Russo C, Buda A, Barresi G, Corsonello A, Lattanzio F, Lorello G, Filippelli G, Marino S (2023). Toll-like receptors and NLRP3 inflammasome-dependent pathways in Parkinson’s disease: mechanisms and therapeutic implications. Journal of Neurology.

[ref-148] Stempelj M, Kedinger M, Augenlicht L, Klampfer L (2007). Essential role of the JAK/STAT1 signaling pathway in the expression of inducible nitric-oxide synthase in intestinal epithelial cells and its regulation by butyrate. Journal of Biological Chemistry.

[ref-149] Stilling RM, van de Wouw M, Clarke G, Stanton C, Dinan TG, Cryan JF (2016). The neuropharmacology of butyrate: the bread and butter of the microbiota-gut-brain axis?. Neurochemistry International.

[ref-150] St. Laurent R, O’Brien LM, Ahmad ST (2013). Sodium butyrate improves locomotor impairment and early mortality in a rotenone-induced drosophila model of Parkinson’s disease. Neuroscience.

[ref-151] Stoll AC, Kemp CJ, Patterson JR, Kubik M, Kuhn N, Benskey M, Duffy MF, Luk KC, Sortwell CE (2024). Alpha-synuclein inclusion responsive microglia are resistant to CSF1R inhibition. Journal of Neuroinflammation.

[ref-152] Stumpff F (2018). A look at the smelly side of physiology: transport of short chain fatty acids. Pflugers Archiv—European Journal of Physiology.

[ref-153] Sulzer D, Alcalay RN, Garretti F, Cote L, Kanter E, Agin-Liebes J, Liong C, McMurtrey C, Hildebrand WH, Mao X, Dawson VL, Dawson TM, Oseroff C, Pham J, Sidney J, Dillon MB, Carpenter C, Weiskopf D, Phillips E, Mallal S, Peters B, Frazier A, Lindestam Arlehamn CS, Sette A (2017). T cells from patients with Parkinson’s disease recognize α-synuclein peptides. Nature.

[ref-154] Tan J, McKenzie C, Potamitis M, Thorburn AN, Mackay CR, Macia L (2014). The role of short-chain fatty acids in health and disease. Advances in Immunology.

[ref-155] Tang Y, Chen Y, Jiang H, Nie D (2011). Short-chain fatty acids induced autophagy serves as an adaptive strategy for retarding mitochondria-mediated apoptotic cell death. Cell Death and Differentiation.

[ref-156] Tarutani A, Hasegawa M (2019). Prion-like propagation of α-synuclein in neurodegenerative diseases. Progress in Molecular Biology and Translational Science.

[ref-157] Toker L, Tran GT, Sundaresan J, Tysnes O-B, Alves G, Haugarvoll K, Nido GS, Dölle C, Tzoulis C (2021). Genome-wide histone acetylation analysis reveals altered transcriptional regulation in the Parkinson’s disease brain. Molecular Neurodegeneration.

[ref-158] Toulany M, Minjgee M, Saki M, Holler M, Meier F, Eicheler W, Rodemann HP (2014). ERK2-dependent reactivation of akt mediates the limited response of tumor cells with constitutive K-RAS activity to PI3K inhibition. Cancer Biology & Therapy.

[ref-159] Tryphena KP, Nikhil US, Pinjala P, Srivastava S, Singh SB, Khatri DK (2023). Mitochondrial complex I as a pathologic and therapeutic target for Parkinson’s disease. ACS Chemical Neuroscience.

[ref-160] Umeno A, Biju V, Yoshida Y (2017). In vivo ROS production and use of oxidative stress-derived biomarkers to detect the onset of diseases such as Alzheimer’s disease, Parkinson’s disease, and diabetes. Free Radical Research.

[ref-161] Unger MM, Spiegel J, Dillmann K-U, Grundmann D, Philippeit H, Bürmann J, Faßbender K, Schwiertz A, Schäfer K-H (2016). Short chain fatty acids and gut microbiota differ between patients with Parkinson’s disease and age-matched controls. Parkinsonism & Related Disorders.

[ref-162] Usman MW, Gao J, Zheng T, Rui C, Li T, Bian X, Cheng H, Liu P, Luo F (2019). Author correction: macrophages confer resistance to PI3K inhibitor GDC-0941 in breast cancer through the activation of NF-κB signaling. Cell Death and Disease.

[ref-163] van Heesbeen HJ, Smidt MP (2019). Entanglement of genetics and epigenetics in Parkinson’s disease. Frontiers in Neuroscience.

[ref-164] Vasudevan Sajini D, Thaggikuppe Krishnamurthy P, Chakkittukandiyil A, Mudavath RN (2024). Orientin modulates Nrf2-ARE, PI3K/akt, JNK-ERK1/2, and TLR4/NF-kB pathways to produce neuroprotective benefits in Parkinson’s disease. Neurochemical Research.

[ref-165] Wanders D, Graff EC, Judd RL (2012). Effects of high fat diet on GPR109A and GPR81 gene expression. Biochemical and Biophysical Research Communications.

[ref-166] Wang F, Zhou H, Deng L, Wang L, Chen J, Zhou X (2020). Serine deficiency exacerbates inflammation and oxidative stress via microbiota-gut-brain axis in D-galactose-induced aging mice. Mediators of Inflammation.

[ref-167] Wang J, Zhu N, Su X, Gao Y, Yang R (2023). Gut-microbiota-derived metabolites maintain gut and systemic immune homeostasis. Cells.

[ref-168] Watchon M, Robinson KJ, Luu L, An Y, Yuan KC, Plenderleith SK, Cheng F, Don EK, Nicholson GA, Lee A, Laird AS (2024). Treatment with sodium butyrate induces autophagy resulting in therapeutic benefits for spinocerebellar ataxia type 3. FASEB Journal: Official Publication of the Federation of American Societies for Experimental Biology.

[ref-169] Wiley NC, Cryan JF, Dinan TG, Ross RP, Stanton C, Cowan CSM, Leonard BE (2021). Production of psychoactive metabolites by gut bacteria. Modern Trends in Psychiatry.

[ref-170] Wong M (2010). Mammalian target of rapamycin (mTOR) inhibition as a potential antiepileptogenic therapy: from tuberous sclerosis to common acquired epilepsies. Epilepsia.

[ref-171] Wu X, Chen PS, Dallas S, Wilson B, Block ML, Wang C-C, Kinyamu H, Lu N, Gao X, Leng Y, Chuang D-M, Zhang W, Lu RB, Hong J-S (2008). Histone deacetylase inhibitors up-regulate astrocyte GDNF and BDNF gene transcription and protect dopaminergic neurons. International Journal of Neuropsychopharmacology.

[ref-172] Wu Q, Dong J, Bai X, Jiang Y, Li J, Fan S, Cheng Y, Jiang G (2022). Propionate ameliorates diabetes-induced neurological dysfunction through regulating the PI3K/akt/eNOS signaling pathway. European Journal of Pharmacology.

[ref-173] Wu J, Jiang Z, Zhang H, Liang W, Huang W, Zhang H, Li Y, Wang Z, Wang J, Jia Y, Liu B, Wu H (2018). Sodium butyrate attenuates diabetes-induced aortic endothelial dysfunction via P300-mediated transcriptional activation of Nrf2. Free Radical Biology & Medicine.

[ref-174] Wu S, Li C, Huang W, Li W, Li RW (2012). Alternative splicing regulated by butyrate in bovine epithelial cells. PLOS ONE.

[ref-175] Xu J, Ao Y-L, Huang C, Song X, Zhang G, Cui W, Wang Y, Zhang X-Q, Zhang Z (2022a). Harmol promotes α-synuclein degradation and improves motor impairment in Parkinson’s models via regulating autophagy-lysosome pathway. npj Parkinson’s Disease.

[ref-176] Xu R-C, Miao W-T, Xu J-Y, Xu W-X, Liu M-R, Ding S-T, Jian Y-X, Lei Y-H, Yan N, Liu H-D (2022b). Neuroprotective effects of sodium butyrate and monomethyl fumarate treatment through GPR109A modulation and intestinal barrier restoration on PD mice. Nutrients.

[ref-177] Yang L, Wang H, Liu L, Xie A (2018). The role of insulin/IGF-1/PI3K/akt/GSK3β signaling in Parkinson’s disease dementia. Frontiers in Neuroscience.

[ref-178] Yao Y, Li P, Jiang S, Meng XL, Gao H, Yang XL (2024). A mechanism study on the antioxidant pathway of Keap1-Nrf2-ARE inhibiting ferroptosis in dopaminergic neurons. Current Molecular Medicine.

[ref-179] Yasuda Y, Tokumatsu T, Ueda C, Sakai M, Sasaki Y, Norikura T, Matsui-Yuasa I, Kojima-Yuasa A (2024). Ecklonia cava polyphenols have a preventive effect on Parkinson’s disease through the activation of the Nrf2-ARE pathway. Nutrients.

[ref-180] Ying X-D, Wei G, An H (2021). Sodium butyrate relieves lung ischemia-reperfusion injury by inhibiting NF-κB and JAK2/STAT3 signaling pathways. European Review for Medical and Pharmacological Sciences.

[ref-181] Yshii L, Pasciuto E, Bielefeld P, Mascali L, Lemaitre P, Marino M, Dooley J, Kouser L, Verschoren S, Lagou V, Kemps H, Gervois P, de Boer A, Burton OT, Wahis J, Verhaert J, Tareen SHK, Roca CP, Singh K, Whyte CE, Kerstens A, Callaerts-Vegh Z, Poovathingal S, Prezzemolo T, Wierda K, Dashwood A, Xie J, Van Wonterghem E, Creemers E, Aloulou M, Gsell W, Abiega O, Munck S, Vandenbroucke RE, Bronckaers A, Lemmens R, De Strooper B, Van Den Bosch L, Himmelreich U, Fitzsimons CP, Holt MG, Liston A (2022). Astrocyte-targeted gene delivery of interleukin 2 specifically increases brain-resident regulatory T cell numbers and protects against pathological neuroinflammation. Nature Immunology.

[ref-182] Zhang L, Blackwell K, Thomas GS, Sun S, Yeh W-C, Habelhah H (2009). TRAF2 suppresses basal IKK activity in resting cells and TNFalpha can activate IKK in TRAF2 and TRAF5 double knockout cells. Journal of Molecular Biology.

[ref-183] Zhang P, Chen X-B, Ding B-Q, Liu H-L, He T (2018). Down-regulation of ABCE1 inhibits temozolomide resistance in glioma through the PI3K/akt/NF-κB signaling pathway. Bioscience Reports.

[ref-184] Zhang D, Jian Y-P, Zhang Y-N, Li Y, Gu L-T, Sun H-H, Liu M-D, Zhou H-L, Wang Y-S, Xu Z-X (2023). Short-chain fatty acids in diseases. Cell Communication and Signaling: CCS.

[ref-185] Zhang Z, Sun X, Wang K, Yu Y, Zhang L, Zhang K, Gu J, Yuan X, Song G (2021). Hydrogen-saturated saline mediated neuroprotection through autophagy via PI3K/AKT/mTOR pathway in early and medium stages of rotenone-induced Parkinson’s disease rats. Brain Research Bulletin.

[ref-186] Zhang Y, Tang Y, Illes P (2024). Modification of neural circuit functions by microglial P2Y6 receptors in health and neurodegeneration. Molecular Neurobiology.

[ref-187] Zhou Z, Xu N, Matei N, McBride DW, Ding Y, Liang H, Tang J, Zhang JH (2021). Sodium butyrate attenuated neuronal apoptosis via GPR41/gβγ/PI3K/akt pathway after MCAO in rats. Journal of Cerebral Blood Flow & Metabolism.

